# A Study of 3D-Printable Reinforced Composite Resin: PMMA Modified with Silver Nanoparticles Loaded Cellulose Nanocrystal

**DOI:** 10.3390/ma11122444

**Published:** 2018-12-03

**Authors:** Shenggui Chen, Junzhong Yang, Yong-Guang Jia, Bingheng Lu, Li Ren

**Affiliations:** 1 School of Materials Science and Engineering, South China University of Technology, Guangzhou 510641, China; segi-11@163.com (S.C.); yangjz925@163.com (J.Y.); ygjia@scut.edu.cn (Y.-G.J.); bhlu@mail.xjtu.edu.cn (B.L.); 2 National Engineering Research Center for Tissue Restoration and Reconstruction, Guangzhou 510006, China; 3 School of Mechanical Engineering, Dongguan University of Technology, Dongguan 523808, China

**Keywords:** 3D printing, PMMA-CNCs-Ag, composite resin, biocompatibility, antibacterial activity

## Abstract

With the rapid application of light-curing 3D printing technology, the demand for high-performance polymer resins is increasing. Existing light-curable resins often have drawbacks limiting their clinical applications. This study aims to develop a new type of polymethyl methacrylate (PMMA) composite resins with enhanced mechanical properties, high antibacterial activities and excellent biocompatibilities. A series of reinforced composite resins were prepared by mechanically mixing PMMA with modified cellulose nanocrystals (CNCs), which were coated with polydopamine and decorated by silver nanoparticles (AgNPs) via Tollen reaction. The morphology of CNCs-Ag was observed by transmission electron microscopy and the formation of AgNPs on CNCs was confirmed by X-Ray photoelectron spectroscopy analyses. Functional groups in PMMA-CNCs-Ag composites were verified by Fourier Transform infrared spectroscopy (FTIR) spectroscopy. The mechanical assessment and scanning electron microscopy analysis suggested that the evenly distributed CNCs-AgNPs composite effectively improve mechanical properties of PMMA resin. Cytotoxicity assay and antibacterial activity tests indicated excellent biocompatibility and high antibacterial activities. Furthermore, PMMA with CNCs-AgNPs of 0.1 wt.% (PMMA-CNCs-AgNPs-0.1) possessed the most desirable mechanical properties owing to the homogeneous distribution of AgNPs throughout the resin matrix. This specific composite resin can be used as a functional dental restoration material with potential of other medical applications.

## 1. Introduction

In clinical applications, 3D printing technology has been widely used in education, pre-operative training and planning, patient-specific medical implant design and consulting, etc. [1] Applications of 3D printing in dental restorations usually include manufacture of dentures, braces and implants. Commonly used 3D printing materials for dental restoration include metal, ceramic, polymer and composite materials. Among them, the light-curing resin is an important polymer material which can quickly convert from a liquid to a solid under the action of light (ultraviolet or visible light). Light-curable resins with long-lasting color and low cost have been used as important raw materials in dental 3D printing application for years. For example, polymethyl methacrylate (PMMA) is one of the most common commercial materials used for the manufacture of denture bases. However, there are some technical challenges that hinder the application of PMMA, such as large shrinkage, low degree of one-time curing, poor mechanical strength, low bacterial resistance, etc., limiting their clinical applications [2,3,4]. This study aims to increase the mechanical properties of PMMA resin as well as to enhance its antibacterial activity by incorporation of the nanomaterials.

The emergence of nanomaterials provides a new way to improve resins’ performance. The most common approach to increase the rigidity of composite resin is to introduce hard-fillers into soft matrix. Nanofillers are considered to be promising fillers for composite resins due to their high specific surface area and high surface free energy, which enhance the bending strength and fracture toughness of the composite resin effectively. For example, wrinkle-structured mesoporous silica (WMS) fillers have been suggested as promising fillers in reinforcing the mechanical performance of nanocomposite resins [5]. Compared with regular silica particles, WMS with unique spherical cores and a wrinkles extending outward structure can interlock with the resin matrix to form firm micromechanical bonding more effectively [5]. A bioinspired catecholic primer, designed as surface priming on nano-fillers, can effectively improve mechanical properties of composite resin due to bidentate hydrogen bonding of catechol moieties to oxide mineral surfaces [6]. Cellulose nanocrystals (CNCs) obtained by acid hydrolysis of natural cellulose have excellent mechanical properties, high surface area and good biocompatibility [7,8]. In recent studies, CNCs have been used as nanofillers for silk fibroin membranes and to reinforce matrixes for collagen scaffolds [9,10]. In addition, PMMA is usually grafted onto the surface of CNCs to modify the properties of CNCs [11], or the modified CNCs have been dosed into PMMA by solution casting [12,13], and these composites are usually used in applications of packaging, flexible screens and optically transparent films. To the best of our knowledge, the use of CNCs as nanofillers to reinforce PMMA resin in dental 3D printing has not been reported yet. In this study, CNCs were introduced to a PMMA matrix to enhance mechanical properties of composite resins.

In addition to the enhancement of mechanical properties, antibacterial activities are also one of key factors affecting the clinical application of composite resins. Though some dental restorative materials are designed with antibacterial properties, the release of these antimicrobial agents is transient and may degrade mechanical properties of composite resins [14]. In addition, it may cause toxic effects on surrounding tissues if antibacterial agents are excessive. Therefore, it is in high demand to develop dental restorative materials with good biocompatibilities and long-lasting antibacterial properties. CaF_2_ nanoparticles have been incorporated into dental resins to enhance the abilities of bearing stress of composite resin as well as sustaining release of fluoride ions (over 10 weeks) [15]. Similar antibacterial effects have also been found by incorporating TiO_2_ into PMMA resin [4]. Quaternary ammonium compounds (QACs) are also one of the most effective disinfectant agents that are widely used in dental materials [16,17]. Functional nanowires of hydroxyapatite (HA) loaded with silver nanoparticles (AgNPs) showed homogenous dispersion in composite resins, and could serve as an efficient reinforcement for composite resins due to enhanced mechanical properties and high antibacterial activities compared with bare resin [18]. Silver nanoparticles have been reported to possess a greater antibacterial activity and lower toxicity compared to ionic silver [19]. Due to AgNPs having a high surface area to volume ratio, it can achieve a strong antibacterial ability while using a relatively low concentration of AgNPs in the composite resin [20].

This study aimed to develop a new type of composite resin with enhanced mechanical properties, high antibacterial activity and excellent biocompatibility. By achieving this goal, a novel nanocrystalline cellulose-silver (CNCs-Ag) composite is introduced into the PMMA matrix. This new composite resin is expected to have improved mechanical properties and antimicrobial activity compared to pure PMMA resin. The structure and properties of the nanofillers were characterized by analytical and optical tests. The mechanical properties and antibacterial activities of PMMA composite resins with nano-filler of CNCs-AgNPs were also investigated.

## 2. Materials and Methods 

### 2.1. Materials

Dental resins of polymethyl methacrylate (PMMA), NextDent Denture 3+ (Pink) with product code NDBAPI01000, were purchased from NextDent (Soesterberg, Netherlands) and used directly. Dopamine hydrochloride (DA·HCl, ≥99%) and Tris (hydroxymethyl aminomethane) (Tris, ≥99.5%) were purchased from Aladdin (Shanghai, China). Other reagents used in this study, including microcrystalline cellulose silver nitrate (AgNO_3_, ≥99%), hydrochloric acid (HCl, 36.5 wt.%), sodium hydroxide (NaOH, ≥99%), ammonium hydroxide (NH_3_·H_2_O, ≥99%) and glucose (≥99.5%), were purchased from Guangzhou Chemical Reagent Factory (Guangzhou, China) and used without further purification.

### 2.2. Synthesis of CNCs-AgNPs

**Scheme 1:**
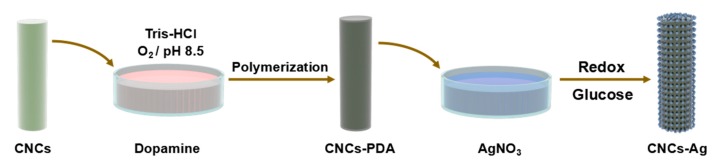
Synthetic route of nanocrystalline cellulose-silver (CNCs-Ag) composite.

The construction of CNCs-AgNPs nanocomposite was based on protocols reported previously [4,18], as illustrated in Scheme 1. Briefly, microcrystalline cellulose powder (10 g) was added to 65% sulfuric acid (90 mL) and the mixture was stirred for 5 h at 55 °C. The mixture was then diluted five-fold to quench the hydrolysis reaction. After cooling to room temperature naturally, the sample was ultrasonically dispersed for 15 min at a frequency of 45 kHz. It was then centrifuged at 5000 rpm for 10 min and the resulting suspension was washed with distilled water three times followed by ultrasonically treating of 45 kHz for 15 min. The above ultrasonication and centrifugation processes were repeated three times. The final dispersion was dialyzed (Viskase 3500 MWCO dialysis membrane) against distilled water till the solution pH reached 7.0. The dialysis continued for one week. Finally, the dialyzed dispersion was left at 4 °C overnight, followed by freeze-drying to get white CNCs.

Before loading AgNPs, CNCs were modified via the oxidized self-polymerization of dopamine to generate an active polydopamine (PDA) coating. Freshly prepared CNCs of 0.2 g was added into 100 mL Tris solution (pH 8.5, 10 mM). Dopamine hydrochloride was then added into Tirs-HCl with a concentration of 2 mg/mL. An aqueous ammonia solution (26%) was added dropwise to AgNO_3_ solution (0.01 M) till the solution was clear and transparent to obtain the silver ammonia solution. CNCs were then dispersed into the prepared dopamine solution (w:v = 1:100), and slowly mixed with 1% silver ammonia solution. Additional glucose (glucose: Ag^+^ = 0.6 in mole) served as a reduction reagent and was added into the above mixture. The solution was stirred at room temperature for 8 h to obtain CNCs loaded with silver nanoparticles for the need of the following procedures, labeled as CNCs-AgNPs.

### 2.3. Preparation of PMMA-CNCs-Ag Composite Resin

A series of PMMA-CNCs-Ag resin (PMMA-CNCs-Ag-0, PMMA-CNCs-Ag-0.05, PMMA-CNCs-Ag-0.1, PMMA-CNCs-Ag-0.15, PMMA-CNCs-Ag-0.2, PMMA-CNCs-Ag-0.25) was prepared with mass fractions of CNCs-Ag to PMMA of 0%, 0.05%, 0.1%, 0.15%, 0.2%, 0.25% respectively. The procedure was briefed as follows: The CNCs-Ag nanocomposite was mechanically stirred for 2 h and ultrasonically treated at 45 Hz for 30 min to obtain a uniform dispersion. PMMA resin was then added into the dispersion and mechanically stirred at 500 rpm/min for 1 h, followed by ultrasonic treatment for 1 h. The bubbles were removed by vacuum to obtain well-dispersed nano-composite resin materials ready for the 3D printing process. 

### 2.4. Characterizations of PMMA-CNCs-Ag Composite Resins

#### 2.4.1. Transmission Electron Microscopy (TEM) Examination

The morphologies of CNCs and CNCs-Ag were characterized by TEM. Drops of aqueous dispersions of samples (0.01%, w/v) were deposited on the carbon-coated electron microscope grids (Protochips Inc., Morrisville, NC, USA) and air dried at room temperature. The grids were observed in a Hitachi HF-2000 transmission electron microscope (Hitachi, Tokyo, Japan). operated at an accelerating voltage of 20 kV.

#### 2.4.2. Fourier Transform Infrared Spectroscopy (FTIR) Analysis

FTIR analysis was performed to identify functional groups present in PMMA-CNCs-Ag composites. The infrared spectra of various PMMA-CNCs-Ag composites were obtained using a Vertex 70 FTIR spectrometer (Bruker, Karlsruhe, Germany). The spectra were recorded in the absorbance mode using a diamond crystal plate, and spectra were obtained at a resolution of 4 cm^−1^ in the spectral region of 500–4000 cm^−1^. Each sample was scanned 20 times.

#### 2.4.3. X-Ray Photoelectron Spectroscopy (XPS) Analysis

XPS analysis was used to confirm the formation of AgNPs on the CNCs. Sample powder of 3 mg was wrapped in an aluminum sheet and pressed into tablets. XPS was performed on an ESCA Lab250 XPS spectrometer (Thermo Electron Corporation, Waltham, MA, USA) with an Al K X-ray source (1486.6 eV photons) under vacuum (10^−8^ Torr or lower) using an incidence angle of 45° at a power of 150 W. 

### 2.5. Mechanical Evaluation of PMMA-CNCs-Ag Composite Resins

Mechanical properties of composite resins (PMMA-CNCs-Ag-0/0.05/0.1/0.15/0.2/0.25) were investigated by a universal testing machine (Instron 5967, Boston, MA, USA). Six rectangular-shaped specimens (64 mm × 10 mm × 3.3 mm), which were printed by Digital Light Projection (DLP) Photocuring 3D printing system (Envision Tech, Gladbeck, Germany), were used for a three-point bending test (span distance 50 mm, cross-head speed 5 mm/min) according to the standard of YY 0270.1-2011/ISO 20795-1:2008 Dentistry-Base polymers-Part 1:Denture base polymers [21].

Fracture surfaces of composite resins after the three-point bending test were observed by S-4800 SEM (Hitachi, Tokyo, Japan). Samples were soaked in ethanol with 80 W ultrasonic cleaner to remove residues on the cross section prior to Gold (Au) coating. The distribution of CNCs-Ag in PMMA matrix across the fracture section was explored through silver element mapping, under the same SEM observation settings with an exposure time of 180 s.

### 2.6. Cytotoxicity Assay

The cytotoxicity of denture base composites was tested by CCK-8 method. The cytotoxicity experiment was performed in triplicate. L929 fibroblasts (provide by Jinan University, Guangzhou, China) were grown in Dulbecco’s modified Eagle’s medium (DMEM, Hyclone) supplemented with 10% fetal bovine serum (FBS, Life technologies), 100 IU/mL penicillin (Sigma, Kawasaki, Japan) and 100 mg/mL streptomycin (Sigma). The culture was maintained with 5% CO_2_ at 37 °C and saturated humidity till 80% confluence before use. According to ISO 10993-12:200, PMMA-CNCs-Ag resin samples were immersed in DMEM (10 mL) for 24 h, and the extracts used for cell culture (1 × 104) were seeded into each well of a 48-well plate. Cells were incubated with various extracts at 37 °C in a humidified atmosphere containing 5% CO_2_. The media were refreshed every two days. The cell proliferation was analyzed using Cell Counting Kit-8 (CCK-8, Beyotime, Shanghai, China). Briefly, at days of 1st, 3rd, 5th, and 7th, 20 L of CCK-8 solution was added into each well and incubated at 37 °C for 4 h, and then the optical density (OD) values were measured using a microreader (Bio-Rad 680) at a wavelength of 450 nm. The OD values were proportional to the number of cells, and cell proliferation curves were plotted by using OD values against incubation time.

### 2.7. Antibacterial Activity

Antibacterial activities of various PMMA-CNCs-Ag composite resins were evaluated using pathogenic bacterial strains of *Staphylococcus aureus* and *Escherichia coli* (provided by Jinan University, Guangzhou, China), and the tests were performed in triplicate. The calibration curve of the relationship between bacterial concentration and OD values were constructed using linear regression. PMMA-CNCs-Ag and bacteria were co-cultured for 24 h, followed by the measurement of the OD value of the bacterial solutions. The corresponding bacterial concentration was calculated using the calibration curve of measured OD values to assess antibacterial effects of PMMA-CNCs-Ag composites.

### 2.8. 3D Printing Trials

A complete denture prototype was manufactured using a Vida model of Digital Light Projection (DLP) Photocuring 3D printing system (Envision Tech, Gladbeck, Germany) to evaluate the compatibility of freshly prepared reinforced composite resin (PMMA-CNCs-Ag-0.1). Firstly, a DL-100 intraoral scanner (Guangdong Langcheng Medical Device Technology Co. Ltd., Shenzhen, China) was used to scan a functional model of maxillary fully edentulous arch. A complete maxillary denture base was designed using a 3D software (3shape, Perfactory Software Suite 3.1, Dearborn, MI, USA) based on the scanned data, which were converted to a STL file and sent to the DLP light-curing 3D printer. The relevant molding parameter settings and slicing processing were applied; the driving codes for the printer were generated, prior to printing. Then, the printer started working when it received the codes. The molding platform was moved up with a distance of 3–4 mm to ensure the separation of the cured layer from the resin tank as well as to facilitate the diffusion of uncured liquid resin to the bottom of molding platform, till the final molding was completed.

### 2.9. Statistical Analyses 

All the results are expressed as mean ± standard deviation (SD). Experiments were repeated three times unless otherwise specified. One-way ANOVA was performed to analyze the variables (Supplemental Materials), and *p* < 0.05 was considered statistically significant.

## 3. Results

### 3.1. Characterizations of CNCs-Ag Composite

**Figure 1:**
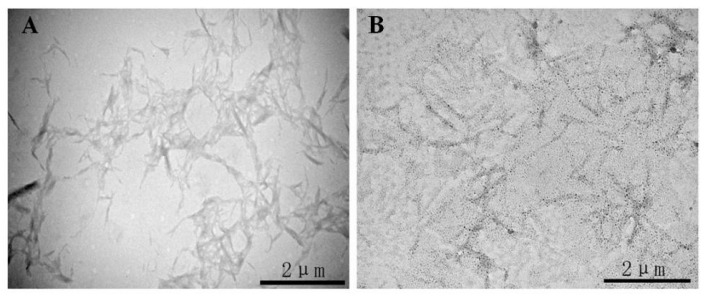
Transmission electron microscopy (TEM) pictures of CNCs-Ag composite: cellulose nanocrystals (CNCs) (**A**); CNCs-Ag (**B**).

**Figure 2:**
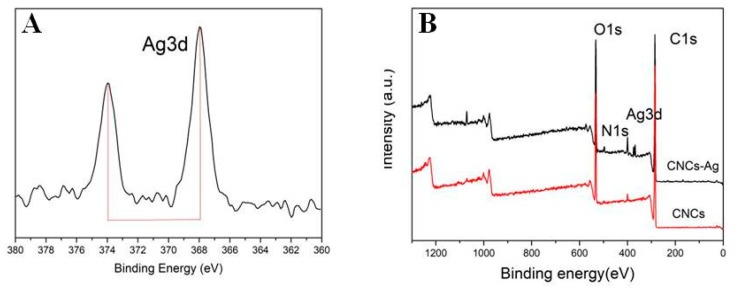
X-ray photoelectron spectroscopy (XPS) analysis of CNCs-Ag composite: Binding energy of Ag_3d_ (**A**); binding energy of CNCs-Ag and CNCs (**B**).

The morphology of CNCs-Ag composites was characterized using TEM, as shown in Figure 1. TEM images showed that the CNCs prepared by acid hydrolysis (Figure 1A) were elongated rods with an average length of 1 m and an average width of 80 nm. Some CNCs were not completely isolated, and had a tendency to aggregate due to the presence of a large number of polar hydroxyl bonds in the cellulose chains. The CNCs were then modified with dopamine which has high surface bonding properties to improve the dispersion of CNCs. CNCs were dispersed in a 2 mg/mL dopamine solution [22] and reacted with Toulon reagent to obtain CNCs-Ag. Numerous uniform nanoparticles appeared on the surface of CNCs (Figure 1B), indicating AgNPs were generated and attached to the CNCs. The XPS analyses further confirmed the formation of AgNPs on CNCs, as shown in Figure 2. The Ag_3d_ signals consisted of two individual peaks at 368 eV and 374 eV, with a spin-orbit separation of 6.0 eV (Figure 2A). These two peaks of 368 eV and 374 eV were the characteristic signals of Ag^0^ referring to the binding energies of Ag_3d3/2_ and Ag_3d5/2_, respectively. In the spectrum of CNCs-Ag (Figure 2B), the Ag_3d_ signals were identified near the site of 370 eV, indicating the presence of Ag in the composite. 

### 3.2. FTIR Analysis of PMMA-CNCs-Ag Composites

**Figure 3:**
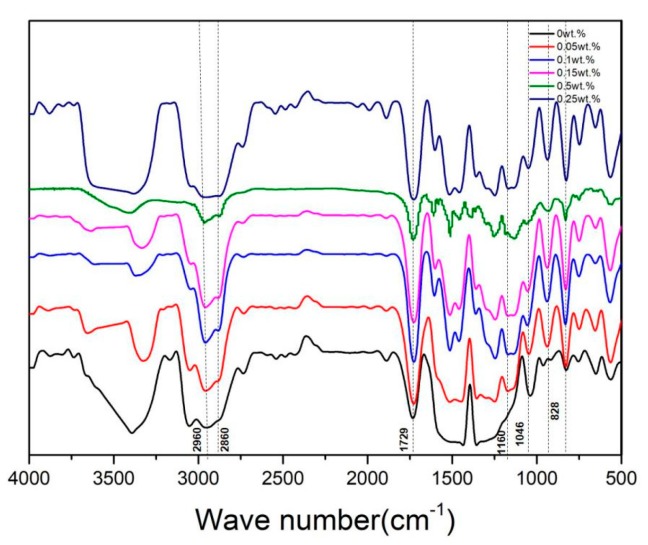
Fourier Transform infrared spectroscopy (FTIR) spectra of polymethyl methacrylate (PMMA) resin containing CNCs-Ag with different mass concentrations: 0%; 0.05%; 0.1%; 0.15%; 0.2% and 0.25%.

A small number of sample powders were prepared by using a file to pry 3D printed resin strips (64 mm × 10 mm × 3.3 mm), and then the KBr tablet method was used, followed by FTIR measurements. In Figure 3, the spectrum of untreated PMMA (0 wt.% CNCs-Ag) showed an absorption band at 1450 cm^−1^, which was attributable to the bending vibration of C-H bond from CH_3_-group. The absorption bands at 2960 cm^−1^ and 2860 cm^−1^ were attributable to the stretching vibration of C-H bond of CH_3_- and CH_2_- groups, respectively. Other characteristic vibration bands included the C=O at 1729 cm^−1^, the ester-based C-O vibration zone at 1046 cm^−1^ and the C-C stretch bands at 961 and 828 cm^−1^. All the bands composed the fingerprint of the PMMA spectrum [23]. With the increase of the content of CNCs-Ag in the composite, the spectrum showed an absorption band from 1160 cm^−1^ to 1250 cm^−1^, which was attributable to the tensile vibration of C-O-C in the PMMA-CNCs-Ag composites. These data suggested that CNCs-Ag composite be successfully attached to PMMA resin.

### 3.3. Mechanical Evaluation of PMMA-CNCs-Ag Composites

**Figure 4:**
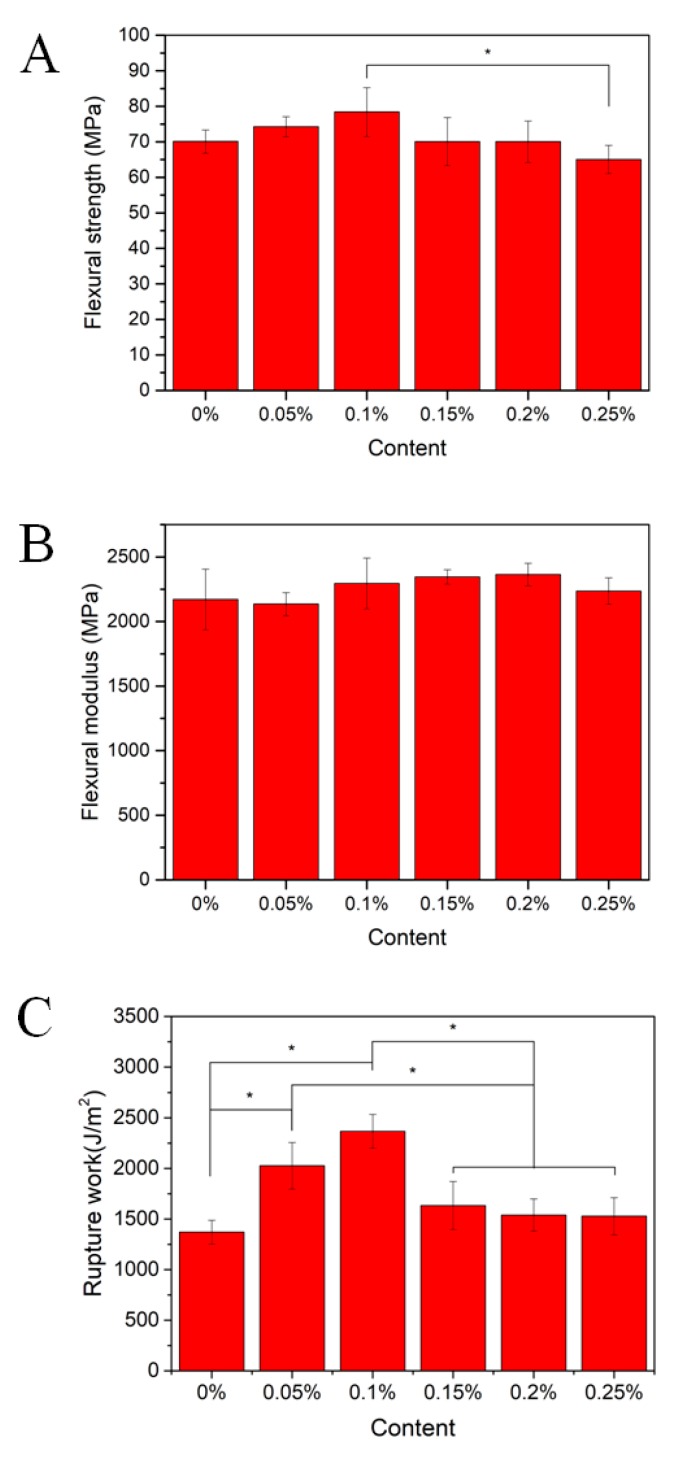
Flexural properties and impact resistance of PMMA resin containing CNCs-Ag with different mass concentrations: (**A**) Flexural strength; (**B**) flexural modulus; (**C**) rupture work.

The flexural strengths of PMMA composite resins increased with the increase of CNCs-Ag content in general (0–0.1 wt.%). As shown in Figure 4A, the flexural strengths of composites modified with 0.05 and 0.1 wt.% CNCs-Ag were increased by approximately 6% and 12%, respectively, compared to a pure resin (PMMA-CNCs-Ag-0%). The flexural strengths of composite resin were not showing further enhancement when the filler contents reached 0.15 and 0.2 wt.%, and in fact the strength was similar to that of the pure resin. When the filler reached 0.25 wt.%, the flexural strength of composite resin dropped to 65.01 ± 3.99 MPa, which was about 8% lower than that of the pure resin. 

The flexural modulus of composite resin also showed a slight increase and then decreased with the further increasing of the filler content (Figure 4B). The flexural modulus of the PMMA composite resin increases with the increase of CNCs-Ag content between 0.1 and 0.2 wt.%. For example, the flexural modulus of the PMMA-CNCs-Ag-0.2 composite resin was 2364 ± 86.6 MPa, which is about 8.8% higher than that of the pure resin. However, when the CNCs-Ag reached 0.25 wt.%, the flexural modulus of the composite resin was 2235.62 ± 102.8 MPa, which is only 3% higher than that of the pure resin. 

The impact strength of PMMA-CNCs-Ag resin was tested by a penetrator, through which the resistance of resin to crack propagation and brittle fracture were analyzed. As shown in Figure 4C, the rupture work of PMMA-CNCs-Ag-0 composite resin was 1370.2 ± 118 J/m^2^. Compared to the PMMA-CNCs-Ag-0 composite resin, the rupture work of other PMMA composites were all increased by adding a certain amount of CNCs-Ag up to 0.25 wt.%. The rupture works of the composites modified with 0.05% and 0.1 wt.% CNCs-Ag reached to 2023.8 ± 229 J/m^2^ and 2367.1 ± 167 J/m^2^, respectively, showing a significant increase of approximately 47.7% and 72.8%, compared to the pure resin. 

### 3.4. Fracture Surface Analysis of PMMA-CNCs-Ag Composites

**Figure 5:**
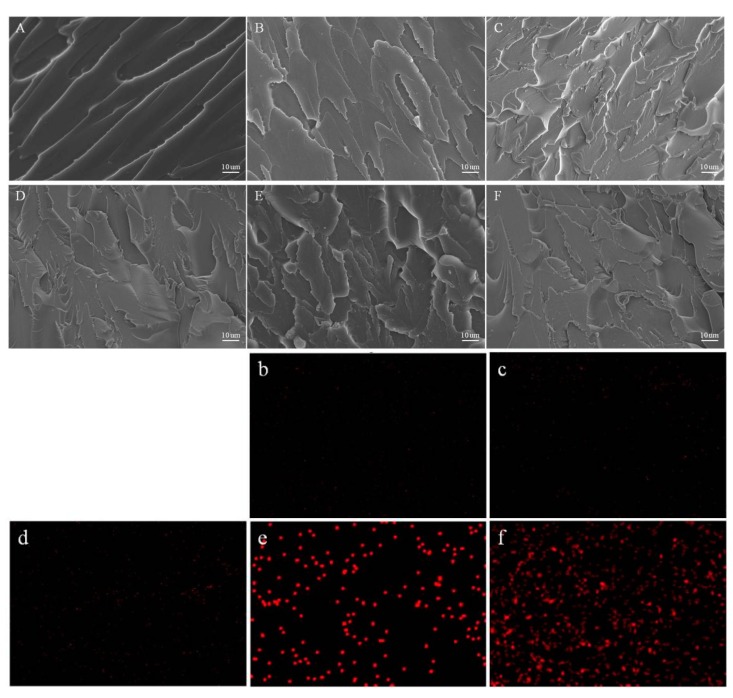
SEM images of the fractured surfaces of PMMA resin containing CNCs-Ag with different mass concentrations after flexural testing: (**A**) 0%; (**B**) 0.05%; (**C**) 0.1%; (**D**) 0.15%; (**E**) 0.2% and (**F**) 0.25%. Panels (**b**–**f**) are the element mapping images of silver element in the resins.

To confirm the dispersion of CNCs-Ag composite in PMMA resins, the fracture cross sections of resin samples were observed by SEM after three-point bending tests. As shown in Figure 5, the cross section of pure PMMA resin was smooth, exhibiting typical brittle fracture characteristics (Figure 5A). This was due to the fact that PMMA was in a glassy state at room temperature and the segment experienced brittle fracture caused by external force. The cross-section surfaces of the other samples with additional CNCs-Ag were much rougher than that of the pure PMMA resin, while small amounts of CNCs-Ag composite were visible in the cross-sectional areas. The Ag element map further revealed the dispersion state of CNCs-Ag in the denture base resin (Figure 5b–f). Ag element signals were observed as scattered and evenly distributed in the samples of PMMA-CNCs-Ag-0.05, PMMA-CNCs-Ag-0.1 and PMMA-CNCs-Ag-0.15. This result indicated that CNCs-Ag did not aggregate in these three samples, as high-density Ag element signals were rarely detected. There were significant high-density Ag element signals observed in the samples of PMMA-CNCs-Ag-0.2 and PMMA-CNCs-Ag-0.25. 

### 3.5. Cytotoxicity of PMMA-CNCs-Ag Composites

**Figure 6:**
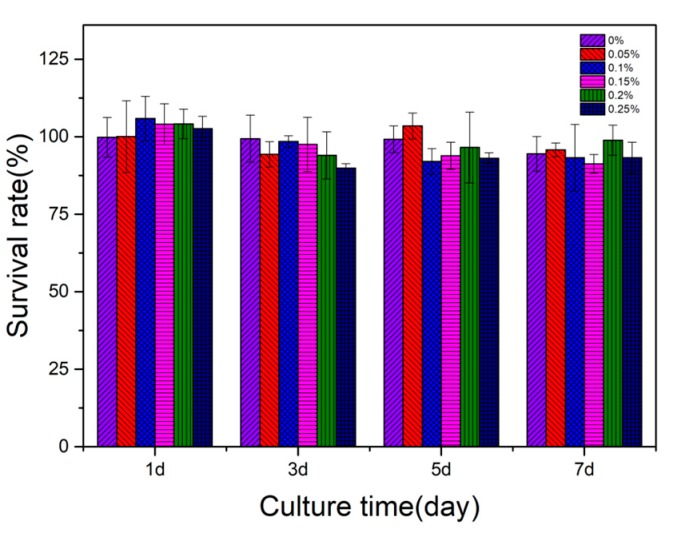
Cytotoxicity assay by culturing L929 fibroblasts in the extracts from PMMA resin containing CNCs-Ag with different mass concentrations, according to ISO 10993-12:200.

The cytotoxicity of various PMMA-CNCs-Ag composites was evaluated by the proliferation of L929 fibroblasts co-culturing with the extract, according to ISO standard 10993-12:200. As shown in Figure 6, PMMA-CNCs-Ag showed almost no toxicity to L929 cells compared with the negative control group. Though the survival rate of L929 cells decreased slightly with time, the reduced survival rate might be caused by rapid proliferation and accumulation of cells in a short time [24]. Overall the cell survival rate for all samples was higher than 85%. As the content of CNCs-Ag in PMMA resin increased, the cell survival rate did not decrease dramatically (*p* > 0.05). This indicated that the addition of a certain amount of CNCs-Ag (0.05–0.25 wt.%) exerted negligible adverse effects on the vitality of L929 fibroblasts.

### 3.6. Antibacterial Activity of PMMA Composites

**Figure 7:**
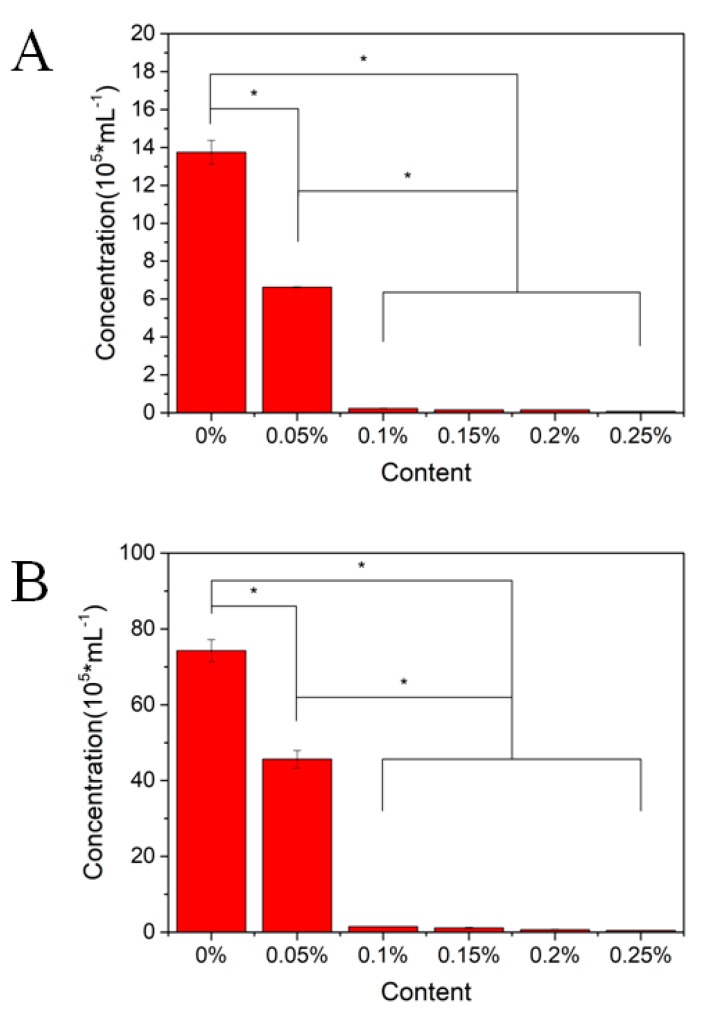
Antibacterial activity of PMMA resin containing CNCs-Ag with different mass concentrations. The composites were co-culturing with: (**A**) *Staphyloccocus aureus*; (**B**) *Escherichia coli*.

In this study, *S. aureus* (Gram-positive bacteria) and *E. coli* (Gram-negative bacteria) were selected as experimental strains to evaluate antibacterial activities of various PMMA-CNCs-Ag composite resins (Figure 7). After incubating the composite samples with the bacteria for 24 h, the concentrations of *S. aureus* and *E. coli* were almost halved for the PMMA-CNCs-Ag-0.05 sample compared to the control group. For other PMMA-CNCs-Ag samples (0.10–0.25 wt.%), the concentrations of both bacteria were significantly reduced to the baseline (*p* < 0.05), indicating that the composite resins have excellent antibacterial effects. The overall performance of the optimal composite resin needs to be considered by the combination of antibacterial and mechanical results before drawing conclusions.

### 3.7. 3D Printing Outcome

**Figure 8:**
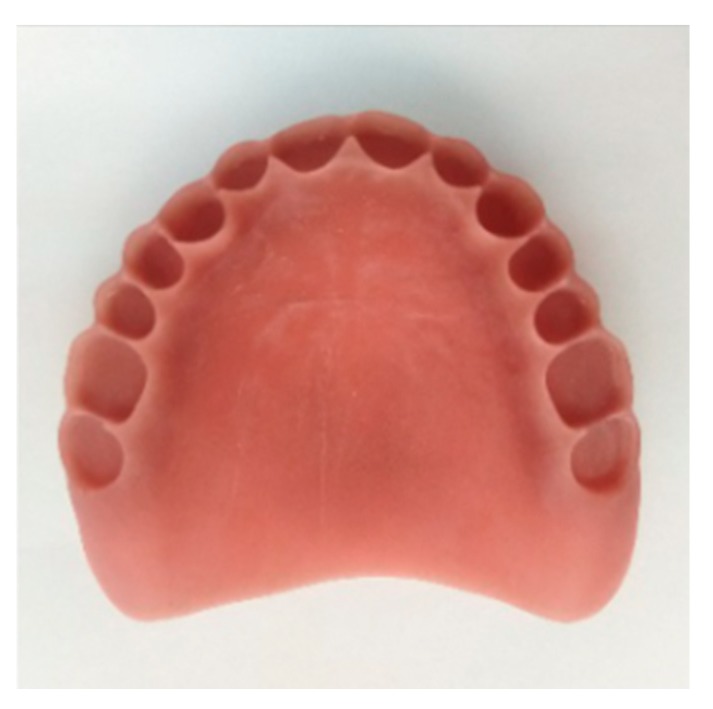
3D printed denture base using composite PMMA containing 1 wt.% CNCs-Ag.

Based on the mechanical evaluation, cytotoxicity and antibacterial activity results, PMMA-CNCs-Ag-0.1 composite resin was selected for the denture base manufacturing using a Vida model by DLP photocuring 3D printing. The printing parameters were set to a thickness of 100 μm for Z-axis layer, a single layer exposure time of 4.4 s, and an ultraviolet light intensity of 2500 μm/cm^2^. The ultraviolet light-curing composite resin was projected by the light machine when the printer was in operation. After each layer was cured, the printing platform was moved upward with the cured product, while the uncured liquid composite resin flowed back to the bottom. The printing platform was then lowered to a position 100 μm from the groove for the next layer of printing. The process was repeated until the dental base was complete. At the end of printing, the base was cleaned with a 95% ethanol solution, dried, removed support, polished and cured using a photocuring box, and finally removed to obtain the final base model (Figure 8).

## 4. Discussion

AgNPs have been shown to provide antibacterial activity for dental composite resins [18,20,25,26]. In this study, the dopamine solution-coated CNCs were reacted with Toluon reagent to form AgNPs in situ, and the uniform dispersion of AgNPs in the composite was successfully achieved, as shown in Figure 1 and Figure 2. FTIR analysis identified the functional groups in PMMA-CNCs-Ag composites, providing molecular structural information for the composites synthesized with different ratios of CNCs-Ag to PMMA (Figure 3). These findings suggested that a series of reinforced composite resins of PMMA-CNCs-Ag were successfully prepared. 

A denture base must sustain appropriate mechanical properties to support its function; fillers are primarily used to improve physical and mechanical properties of the composite resin [27,28]. In this study, three-point bending tests were conducted to investigate the effect of different amounts of CNCs-Ag on mechanical properties of PMMA composites. The results are showed in Figure 4. It indicated that the addition of CNCs-Ag could not serve as a constant strengthener to the PMMA composite resin, but there is a critical and optimal value. This might be due to the fact that the CNCs-Ag composite has a high surface area to volume ratio. Although the interfaces between CNCs-Ag and PMMA resin were well bonded, the large specific surface area of nanofillers might cause aggregation between themselves when the addition of CNCs-Ag reached the critical value. The aggregation may result in the decrease in mechanical properties of the PMMA-CNCs-Ag composite. 

The brittleness of pure PMMA resin may cause cracks in the denture base and shorten its service life. The mechanical properties of PMMA-CNCs-Ag composites were improved, especially the impact resistance, by adding nanofiller CNCs-Ag in the resin. It indicated that the addition of CNCs-Ag nano-composites can effectively improve the impact resistance of PMMA resin. This might be due to the mechanical yielding of the polymer matrix around the PMMA resin when resins were subjected to a severe impact. CNCs-Ag nano-composites could absorb a large amount of deformation work, which might effectively hinder the development of cracks in the resin as well as prevent further crack propagation. In addition, the interface between CNCs-Ag and PMMA resin might be partially debonded, which might generate voids to prevent small cracks from expanding into destructive cracks, resulting in a toughening effect on the PMMA composite resin. This study has shown an optimal CNCs-AgNPs content with regard to the improvement of PMMA resistance, and has found that a further increase of nano-scaled contents in resins may cause aggregation in the system, and thus mechanical properties of composite resin are degraded [18,29].

The distribution map of Ag served as an indicator for analyzing the distribution of CNCs-Ag in PMMA composite resin. Results (Figure 5) suggested that CNCs-Ag composite may aggregate in the samples of PMMA-CNCs-Ag-0.2 and PMMA-CNCs-Ag-0.25. The aggregation trend increased with the addition of CNCs-Ag composite. This is consistent with the analyses of mechanical properties of composite resins, as well as the observations from the SEM analyses of cross-sections. Other studies reported similar trends, as the mechanical properties of composite resins decline with increasing nano-additives [18,29]. This is because an increase in the content of the nano-additive, makes it easier to aggregate in the resin matrix. A study has revealed that compared with 0.1% and 0.2% additions, a reduced amount (0.05%) of nano-silica was more useful in improving mechanical properties of composite resins [29].

Numerous studies have been conducted to improve antibacterial properties for dental restoration materials, such as fluorides [15,30,31], zinc ions [32,33,34,35], silver nanoparticles and silver ions [18,20,25,26,36], and quaternary ammonium groups [25,26,37] are introduced into the fillers through different reaction mechanisms to enhance antimicrobial functions of composite resins. In this study, as the silver content in the resin matrix increased, the antibacterial effect of the resin became more and more significant. PMMA-CNCs-Ag-0.1 composite resin can inhibit more than 95% of *S. aureus* (Gram-positive bacteria) and *E. coli* (Gram-negative bacteria) growth. Further increase in the content of AgNPs, the antibacterial effect is similar to that of PMMA-CNCs-Ag-0.1 composite resin. In addition, cytotoxicity studies (Figure 6) showed that a certain amount of CNCs-Ag (0.05–0.25 wt.%) had no significant effect on the activity of L929 fibroblasts.

Here, we chose PMMA-CNCs-Ag-0.1 composite resin for the optimal composite resin, taking into account the combination of cytotoxic, antibacterial and mechanical results. PMMA-CNCs-Ag-0.1 composite resins can be used to customize temporary or long-term use of restorations with better mechanical and antimicrobial properties when compared to conventional resins. Further studies are required to assess the mechanical resistance of PMMA-CNCs-Ag composite resins in weak acid environments as well as characterize conditions of maintenance in long-term use, for a better clinical application of 3D printed dentures.

## 5. Conclusions

The reinforced composite resins of PMMA-CNCs-Ag were successfully prepared in this study. Structural and morphological analyses (with FTIR and SEM) suggested an optimal range of CNCs-Ag (0–0.15 wt.%) as a nano-filler with good dispensability in a PMMA resin matrix. Composite resin (CNCs-Ag 0.10–0.25 wt.%) exhibit high antibacterial activity with no significant cytotoxic effect. Incorporated with the mechanical tests, PMMA-CNCs-Ag-0.1 composite resin shows great potential and is recommended as a functional dental restoration material. Future work will focus on the optimization of printing parameters using the selected reinforced composite resins, with the purpose of improving the accuracy of 3D printed dental materials. The application of printed denture base manufactured with the studied composite resins will also be evaluated, in particular in dental clinical trials.

## Supplementary Materials

The following are available online at http://www.mdpi.com/1996-1944/11/12/2444/s1.
