# Supplementary material for: A Study of 3D-Printable Reinforced Composite Resin: PMMA Modified with Silver Nanoparticles Loaded Cellulose Nanocrystal
Source: Materials (Basel). 2018 Dec 3;11(12):2444. doi: 10.3390/ma11122444 (PMC6317163; doi:10.3390/ma11122444)
Supplement: Supplementary file 1 [file materials-11-02444-s001.zip › anova fig4&7.pdf]

```
SET BASETEXTDIRECTION=AUTOMATIC TABLERENDER=light ROWSBREAK=100
SUMMARY=None TOLERANCE=1 CELLSBREAK=10000 TLook=None Printback=On
Locale=OSLOCALE MIOUTPUT=[observed imputed pooled diagnostics] OLang=English
Unicode=Yes TFit=Both.
ONEWAY VAR00001 BY CNCsAg
  /STATISTICS DESCRIPTIVES HOMOGENEITY
  /PLOT MEANS
  /MISSING ANALYSIS
  /POSTHOC=BONFERRONI ALPHA(0.05).
```

Oneway

Notes

Output Created		22-NOV-2018 11:30:29
Comments		
Input	Data	C:\Users\Beogene\Desktop\2.sav
	Active Dataset	
	Filter	<none>
	Weight	<none>
	Split File	<none>
	N of Rows in Working Data File	36
Missing Value Handling	Definition of Missing	User-defined missing values are treated as missing.
	Cases Used	Statistics for each analysis are based on cases with no missing data for any variable in the analysis.
Syntax		ONEWAY VAR00001 BY CNCsAg /STATISTICS DESCRIPTIVES HOMOGENEITY /PLOT MEANS /MISSING ANALYSIS /POSTHOC=BONFERRONI ALPHA(0.05).
Resources	Processor Time	00:00:00.49
	Elapsed Time	00:00:00.65

Descriptives

concentration of S aureus

	N	Mean	Std. Deviation	Std. Error	95% Confidence Interval for Mean	
					Lower Bound	Upper Bound
.00	3	13.738409	.6365656	.3675213	12.157092	15.319725
.05	3	6.618381	.0299828	.0173106	6.543900	6.692863
.10	3	.232922	.0108879	.0062861	.205875	.259969
.15	3	.169822	.0023759	.0013717	.163920	.175724
.20	3	.150617	.0163318	.0094292	.110047	.191188
.25	3	.070370	.0381075	.0220014	-.024294	.165035
Total	18	3.496754	5.3064750	1.2507482	.857906	6.135601

Descriptives

concentration of S aureus

	Minimum	Maximum
.00	13.2535	14.4593
.05	6.5971	6.6527
.10	.2247	.2453
.15	.1671	.1712
.20	.1321	.1630
.25	.0416	.1136
Total	.0416	14.4593

Test of Homogeneity of Variances

concentration of S aureus

Levene Statistic	df1	df2	Sig.
11.026	5	12	.000

ANOVA

concentration of S aureus

	Sum of Squares	df	Mean Square	F	Sig.
Between Groups	477.882	5	95.576	1405.679	.000
Within Groups	.816	12	.068		
Total	478.698	17			

Post Hoc Tests

Multiple Comparisons

Dependent Variable: concentration of S aureus

Bonferroni

(I) CNCsAg	(J) CNCsAg	Mean Difference (I-J)	Std. Error	Sig.	95% Confidence Interval Lower Bound	95% Confidence Interval Upper Bound
.00	.05	7.1200274*	.2129052	.000	6.343160	7.896895
	.10	13.5054870*	.2129052	.000	12.728619	14.282355
	.15	13.5685871*	.2129052	.000	12.791719	14.345455
	.20	13.5877915*	.2129052	.000	12.810924	14.364659
	.25	13.6680384*	.2129052	.000	12.891171	14.444906
.05	.00	-7.1200274*	.2129052	.000	-7.896895	-6.343160
	.10	6.3854595*	.2129052	.000	5.608592	7.162327
	.15	6.4485597*	.2129052	.000	5.671692	7.225427
	.20	6.4677641*	.2129052	.000	5.690896	7.244632
	.25	6.5480110*	.2129052	.000	5.771143	7.324879
.10	.00	-13.5054870*	.2129052	.000	-14.282355	-12.728619
	.05	-6.3854595*	.2129052	.000	-7.162327	-5.608592
	.15	.0631001	.2129052	1.000	-.713768	.839968
	.20	.0823045	.2129052	1.000	-.694563	.859172
	.25	.1625514	.2129052	1.000	-.614316	.939419
.15	.00	-13.5685871*	.2129052	.000	-14.345455	-12.791719
	.05	-6.4485597*	.2129052	.000	-7.225427	-5.671692
	.10	-.0631001	.2129052	1.000	-.839968	.713768
	.20	.0192044	.2129052	1.000	-.757663	.796072
	.25	.0994513	.2129052	1.000	-.677416	.876319
.20	.00	-13.5877915*	.2129052	.000	-14.364659	-12.810924
	.05	-6.4677641*	.2129052	.000	-7.244632	-5.690896
	.10	-.0823045	.2129052	1.000	-.859172	.694563
	.15	-.0192044	.2129052	1.000	-.796072	.757663
	.25	.0802469	.2129052	1.000	-.696621	.857115
.25	.00	-13.6680384*	.2129052	.000	-14.444906	-12.891171
	.05	-6.5480110*	.2129052	.000	-7.324879	-5.771143
	.10	-.1625514	.2129052	1.000	-.939419	.614316
	.15	-.0994513	.2129052	1.000	-.876319	.677416
	.20	-.0802469	.2129052	1.000	-.857115	.696621

*. The mean difference is significant at the 0.05 level.

Means Plots

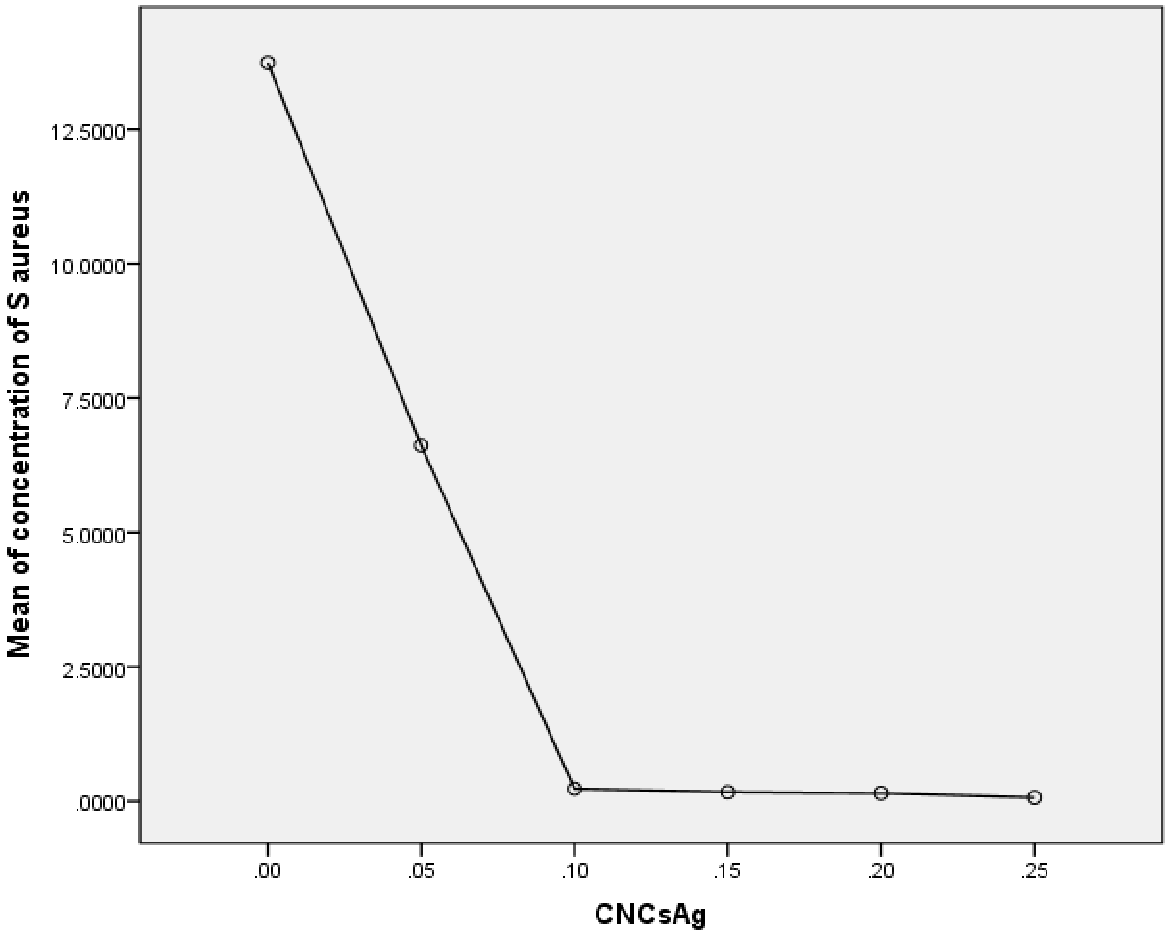


```
ONEWAY VAR00002 BY CNCsAg
  /STATISTICS DESCRIPTIVES HOMOGENEITY
  /PLOT MEANS
  /MISSING ANALYSIS
  /POSTHOC=BONFERRONI ALPHA(0.05).
```

Oneway

Notes

Output Created		22-NOV-2018 11:31:06
Comments		
Input	Data	C:\Users\Beogene\Desktop\ 2.sav
	Active Dataset	
	Filter	<none>
	Weight	<none>
	Split File	<none>
	N of Rows in Working Data File	36
Missing Value Handling	Definition of Missing	User-defined missing values are treated as missing.
	Cases Used	Statistics for each analysis are based on cases with no missing data for any variable in the analysis.
Syntax		ONEWAY VAR00002 BY CNCsAg /STATISTICS DESCRIPTIVES HOMOGENEITY /PLOT MEANS /MISSING ANALYSIS /POSTHOC=BONFERRONI ALPHA(0.05).
Resources	Processor Time	00:00:00.49
	Elapsed Time	00:00:00.61

Descriptives

concentration of E coli

	N	Mean	Std. Deviation	Std. Error	95% Confidence Interval for Mean	
					Lower Bound	Upper Bound
.00	3	74.269113	2.9580340	1.7078217	66.920949	81.617277
.05	3	45.655454	2.3085309	1.3328310	39.920745	51.390162
.10	3	1.476045	.0536985	.0310029	1.342650	1.609439
.15	3	1.150357	.1258135	.0726385	.837819	1.462895
.20	3	.680938	.0784649	.0453017	.486020	.875855

.25	3	.482161	.0088280	.0050968	.460231	.504091
Total	18	20.619011	29.8911581	7.0454135	5.754488	35.483534

Descriptives

concentration of E coli

	Minimum	Maximum
.00	71.8226	77.5566
.05	43.1835	47.7554
.10	1.4251	1.5321
.15	1.0122	1.2584
.20	.6147	.7676
.25	.4771	.4924
Total	.4771	77.5566

Test of Homogeneity of Variances

concentration of E coli

Levene Statistic	df1	df2	Sig.
6.186	5	12	.005

ANOVA

concentration of E coli

	Sum of Squares	df	Mean Square	F	Sig.
Between Groups	15160.974	5	3032.195	1289.909	.000
Within Groups	28.208	12	2.351		
Total	15189.183	17			

Post Hoc Tests

Multiple Comparisons

Dependent Variable: concentration of E coli

Bonferroni

(I) CNCsAg	(J) CNCsAg	Mean Difference (I-J)	Std. Error	Sig.	95% Confidence Interval	
					Lower Bound	Upper Bound
.00	.05	28.6136595*	1.2518532	.000	24.045786	33.181533
	.10	72.7930683*	1.2518532	.000	68.225195	77.360942
	.15	73.1187564*	1.2518532	.000	68.550883	77.686630
	.20	73.5881753*	1.2518532	.000	69.020302	78.156049
	.25	73.7869521*	1.2518532	.000	69.219078	78.354826
.05	.00	-28.6136595*	1.2518532	.000	-33.181533	-24.045786
	.10	44.1794088*	1.2518532	.000	39.611535	48.747282
	.15	44.5050968*	1.2518532	.000	39.937223	49.072970
	.20	44.9745158*	1.2518532	.000	40.406642	49.542389
	.25	45.1732926*	1.2518532	.000	40.605419	49.741166
.10	.00	-72.7930683*	1.2518532	.000	-77.360942	-68.225195
	.05	-44.1794088*	1.2518532	.000	-48.747282	-39.611535
	.15	.3256881	1.2518532	1.000	-4.242186	4.893562
	.20	.7951070	1.2518532	1.000	-3.772767	5.362981
	.25	.9938838	1.2518532	1.000	-3.573990	5.561757
.15	.00	-73.1187564*	1.2518532	.000	-77.686630	-68.550883
	.05	-44.5050968*	1.2518532	.000	-49.072970	-39.937223
	.10	-.3256881	1.2518532	1.000	-4.893562	4.242186
	.20	.4694190	1.2518532	1.000	-4.098455	5.037293
	.25	.6681957	1.2518532	1.000	-3.899678	5.236069
.20	.00	-73.5881753*	1.2518532	.000	-78.156049	-69.020302
	.05	-44.9745158*	1.2518532	.000	-49.542389	-40.406642
	.10	-.7951070	1.2518532	1.000	-5.362981	3.772767
	.15	-.4694190	1.2518532	1.000	-5.037293	4.098455
	.25	.1987768	1.2518532	1.000	-4.369097	4.766650
.25	.00	-73.7869521*	1.2518532	.000	-78.354826	-69.219078
	.05	-45.1732926*	1.2518532	.000	-49.741166	-40.605419
	.10	-.9938838	1.2518532	1.000	-5.561757	3.573990
	.15	-.6681957	1.2518532	1.000	-5.236069	3.899678
	.20	-.1987768	1.2518532	1.000	-4.766650	4.369097

*. The mean difference is significant at the 0.05 level.

Means Plots

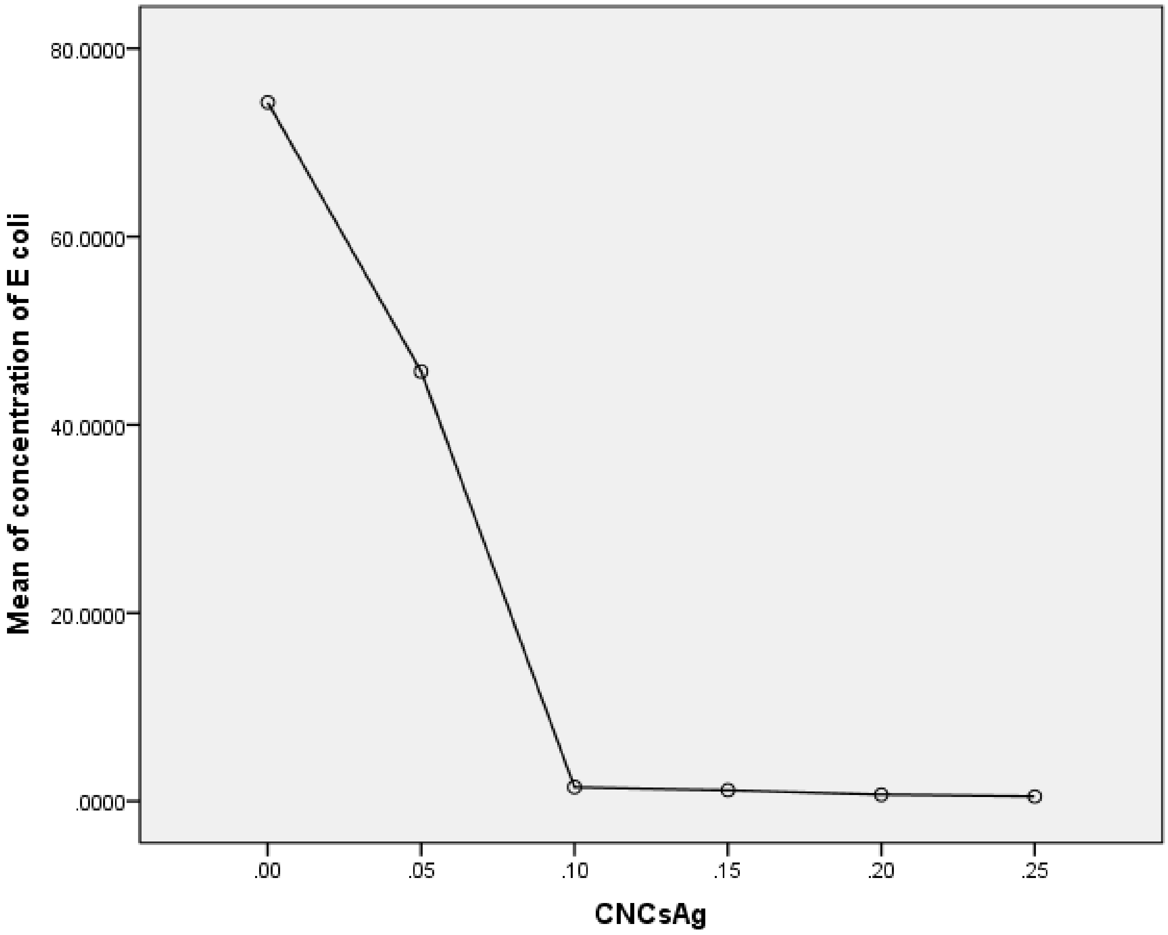

80.0000
60.0000
40.0000
20.0000
.0000
Mean of concentration of E coli
.00
.05
.10
.15
.20
.25
CNCsAg

```
ONEWAY VAR00004 BY VAR00003
  /STATISTICS DESCRIPTIVES HOMOGENEITY
  /PLOT MEANS
  /MISSING ANALYSIS
  /POSTHOC=BONFERRONI ALPHA(0.05).
```

Oneway

Notes

Output Created		22-NOV-2018 11:33:14
Comments		
Input	Data	C:\Users\Beogene\Desktop\ 2.sav
	Active Dataset	
	Filter	<none>
	Weight	<none>
	Split File	<none>
	N of Rows in Working Data File	36
Missing Value Handling	Definition of Missing	User-defined missing values are treated as missing.
	Cases Used	Statistics for each analysis are based on cases with no missing data for any variable in the analysis.
Syntax		ONEWAY VAR00004 BY VAR00003 /STATISTICS DESCRIPTIVES HOMOGENEITY /PLOT MEANS /MISSING ANALYSIS /POSTHOC=BONFERRONI ALPHA(0.05).
Resources	Processor Time	00:00:00.49
	Elapsed Time	00:00:00.55

Descriptives

Rupture work

	N	Mean	Std. Deviation	Std. Error	95% Confidence Interval for Mean	
					Lower Bound	Upper Bound
.00	6	1370.191813	117.7550320	48.0732905	1246.615485	1493.768140
.05	6	2026.806413	229.1728882	93.5594398	1786.304217	2267.308610
.10	6	2367.098153	167.1699281	68.2468373	2191.664073	2542.532234
.15	6	1633.408545	236.8850186	96.7079039	1384.812964	1882.004126
.20	6	1538.102874	160.2182610	65.4088312	1369.964120	1706.241627
.25	6	1528.083469	183.7001310	74.9952644	1335.302004	1720.864934
Total	36	1743.948544	389.3054669	64.8842445	1612.226525	1875.670564

Descriptives

Rupture work

	Minimum	Maximum
.00	1232.6978	1549.6087
.05	1807.2057	2329.8561
.10	2066.3023	2524.2750
.15	1331.5401	1913.1300
.20	1367.6904	1826.6245
.25	1308.2899	1780.6061
Total	1232.6978	2524.2750

Test of Homogeneity of Variances

Rupture work

Levene Statistic	df1	df2	Sig.
1.205	5	30	.331

ANOVA

Rupture work

	Sum of Squares	df	Mean Square	F	Sig.
Between Groups	4255244.196	5	851048.839	24.332	.000
Within Groups	1049311.932	30	34977.064		
Total	5304556.128	35			

Post Hoc Tests

Multiple Comparisons

Dependent Variable: Rupture work

Bonferroni

(I) CNCsAg	(J) CNCsAg	Mean Difference (I-J)	Std. Error	Sig.	95% Confidence Interval Lower Bound
.00	.05	-656.6146005*	107.9769488	.000	-1000.932130
	.10	-996.9063407*	107.9769488	.000	-1341.223870
	.15	-263.2167320	107.9769488	.314	-607.534262
	.20	-167.9110610	107.9769488	1.000	-512.228591
	.25	-157.8916563	107.9769488	1.000	-502.209186
.05	.00	656.6146005*	107.9769488	.000	312.297071
	.10	-340.2917402	107.9769488	.055	-684.609270
	.15	393.3978685*	107.9769488	.015	49.080339
	.20	488.7035395*	107.9769488	.001	144.386010
	.25	498.7229442*	107.9769488	.001	154.405415
.10	.00	996.9063407*	107.9769488	.000	652.588811
	.05	340.2917402	107.9769488	.055	-4.025789
	.15	733.6896087*	107.9769488	.000	389.372079
	.20	828.9952797*	107.9769488	.000	484.677750
	.25	839.0146843*	107.9769488	.000	494.697155
.15	.00	263.2167320	107.9769488	.314	-81.100798
	.05	-393.3978685*	107.9769488	.015	-737.715398
	.10	-733.6896087*	107.9769488	.000	-1078.007138
	.20	95.3056710	107.9769488	1.000	-249.011859
	.25	105.3250757	107.9769488	1.000	-238.992454
.20	.00	167.9110610	107.9769488	1.000	-176.406469
	.05	-488.7035395*	107.9769488	.001	-833.021069
	.10	-828.9952797*	107.9769488	.000	-1173.312809
	.15	-95.3056710	107.9769488	1.000	-439.623201
	.25	10.0194047	107.9769488	1.000	-334.298125
.25	.00	157.8916563	107.9769488	1.000	-186.425873

.05	-498.7229442*	107.9769488	.001	-843.040474
.10	-839.0146843*	107.9769488	.000	-1183.332214
.15	-105.3250757	107.9769488	1.000	-449.642605
.20	-10.0194047	107.9769488	1.000	-354.336934

Multiple Comparisons

Dependent Variable: Rupture work

Bonferroni

		95% Confidence Interval
(I) CNCsAg	(J) CNCsAg	Upper Bound
.00	.05	-312.297071
	.10	-652.588811
	.15	81.100798
	.20	176.406469
	.25	186.425873
.05	.00	1000.932130
	.10	4.025789
	.15	737.715398
	.20	833.021069
	.25	843.040474
.10	.00	1341.223870
	.05	684.609270
	.15	1078.007138
	.20	1173.312809
	.25	1183.332214
.15	.00	607.534262
	.05	-49.080339
	.10	-389.372079
	.20	439.623201
	.25	449.642605
.20	.00	512.228591
	.05	-144.386010
	.10	-484.677750
	.15	249.011859
	.25	354.336934
.25	.00	502.209186
	.05	-154.405415
	.10	-494.697155
	.15	238.992454
	.20	334.298125

*. The mean difference is significant at the 0.05 level.

Means Plots

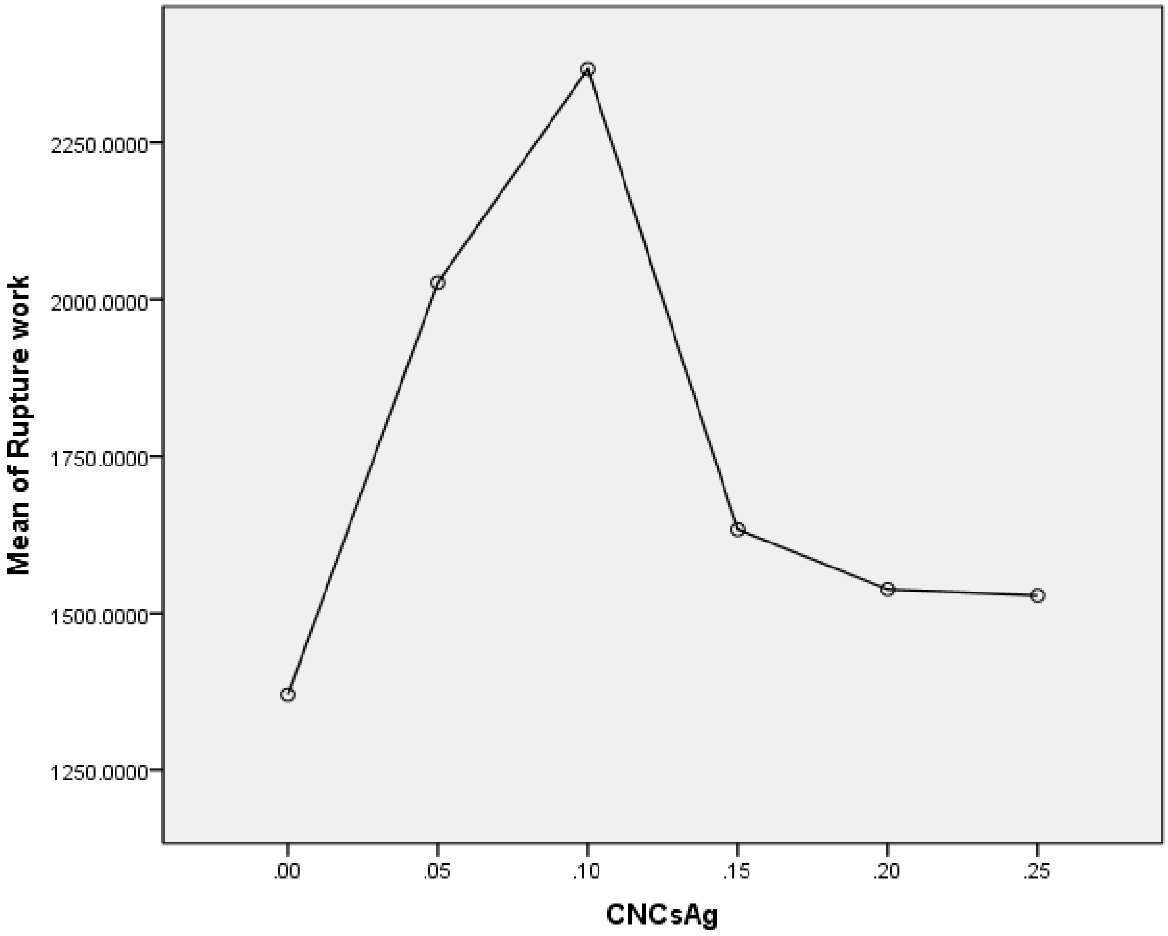


```
ONEWAY VAR00006 BY VAR00005
  /STATISTICS DESCRIPTIVES HOMOGENEITY
  /PLOT MEANS
  /MISSING ANALYSIS
  /POSTHOC=BONFERRONI ALPHA(0.05).
```

Oneway

Notes

Output Created		22-NOV-2018 11:33:51
Comments		
Input	Data	C:\Users\Beogene\Desktop\ 2.sav
	Active Dataset	
	Filter	<none>
	Weight	<none>
	Split File	<none>
	N of Rows in Working Data File	36
Missing Value Handling	Definition of Missing	User-defined missing values are treated as missing.
	Cases Used	Statistics for each analysis are based on cases with no missing data for any variable in the analysis.
Syntax		ONEWAY VAR00006 BY VAR00005 /STATISTICS DESCRIPTIVES HOMOGENEITY /PLOT MEANS /MISSING ANALYSIS /POSTHOC=BONFERRONI ALPHA(0.05).
Resources	Processor Time	00:00:00.64
	Elapsed Time	00:00:00.76

Descriptives

Flexural strength

					95% Confidence Interval for Mean	
	N	Mean	Std. Deviation	Std. Error	Lower Bound	Upper Bound
.00	5	70.0879	3.27879	1.46632	66.0168	74.1591
.05	5	74.2587	2.79423	1.24962	70.7892	77.7281

.10	5	78.3980	6.87760	3.07576	69.8583	86.9376
.15	5	70.0516	6.78503	3.03436	61.6269	78.4763
.20	5	70.0499	5.82054	2.60303	62.8227	77.2770
.25	5	65.0099	3.99535	1.78677	60.0490	69.9708
Total	30	71.3093	6.33382	1.15639	68.9442	73.6744

Descriptives

Flexural strength

	Minimum	Maximum
.00	65.01	73.57
.05	71.45	78.84
.10	70.95	87.42
.15	61.26	77.46
.20	62.09	76.10
.25	60.32	68.48
Total	60.32	87.42

Test of Homogeneity of Variances

Flexural strength

Levene Statistic	df1	df2	Sig.
2.338	5	24	.073

ANOVA

Flexural strength

	Sum of Squares	df	Mean Square	F	Sig.
Between Groups	516.451	5	103.290	3.832	.011
Within Groups	646.951	24	26.956		
Total	1163.402	29			

Post Hoc Tests

Multiple Comparisons

Dependent Variable: Flexural strength

Bonferroni

(I) CNCsAg	(J) CNCsAg	Mean Difference (I-J)	Std. Error	Sig.	95% Confidence Interval	
					Lower Bound	Upper Bound
.00	.05	-4.17072	3.28367	1.000	-14.8702	6.5287
	.10	-8.31002	3.28367	.275	-19.0095	2.3894
	.15	.03632	3.28367	1.000	-10.6631	10.7358
	.20	.03806	3.28367	1.000	-10.6614	10.7375
	.25	5.07806	3.28367	1.000	-5.6214	15.7775
.05	.00	4.17072	3.28367	1.000	-6.5287	14.8702
	.10	-4.13930	3.28367	1.000	-14.8388	6.5602
	.15	4.20704	3.28367	1.000	-6.4924	14.9065
	.20	4.20878	3.28367	1.000	-6.4907	14.9082
	.25	9.24878	3.28367	.143	-1.4507	19.9482
.10	.00	8.31002	3.28367	.275	-2.3894	19.0095
	.05	4.13930	3.28367	1.000	-6.5602	14.8388
	.15	8.34634	3.28367	.269	-2.3531	19.0458
	.20	8.34808	3.28367	.268	-2.3514	19.0475
	.25	13.38808*	3.28367	.007	2.6886	24.0875
.15	.00	-.03632	3.28367	1.000	-10.7358	10.6631
	.05	-4.20704	3.28367	1.000	-14.9065	6.4924
	.10	-8.34634	3.28367	.269	-19.0458	2.3531
	.20	.00174	3.28367	1.000	-10.6977	10.7012
	.25	5.04174	3.28367	1.000	-5.6577	15.7412
.20	.00	-.03806	3.28367	1.000	-10.7375	10.6614
	.05	-4.20878	3.28367	1.000	-14.9082	6.4907
	.10	-8.34808	3.28367	.268	-19.0475	2.3514
	.15	-.00174	3.28367	1.000	-10.7012	10.6977
	.25	5.04000	3.28367	1.000	-5.6595	15.7395
.25	.00	-5.07806	3.28367	1.000	-15.7775	5.6214
	.05	-9.24878	3.28367	.143	-19.9482	1.4507
	.10	-13.38808*	3.28367	.007	-24.0875	-2.6886
	.15	-5.04174	3.28367	1.000	-15.7412	5.6577
	.20	-5.04000	3.28367	1.000	-15.7395	5.6595

*. The mean difference is significant at the 0.05 level.

Means Plots

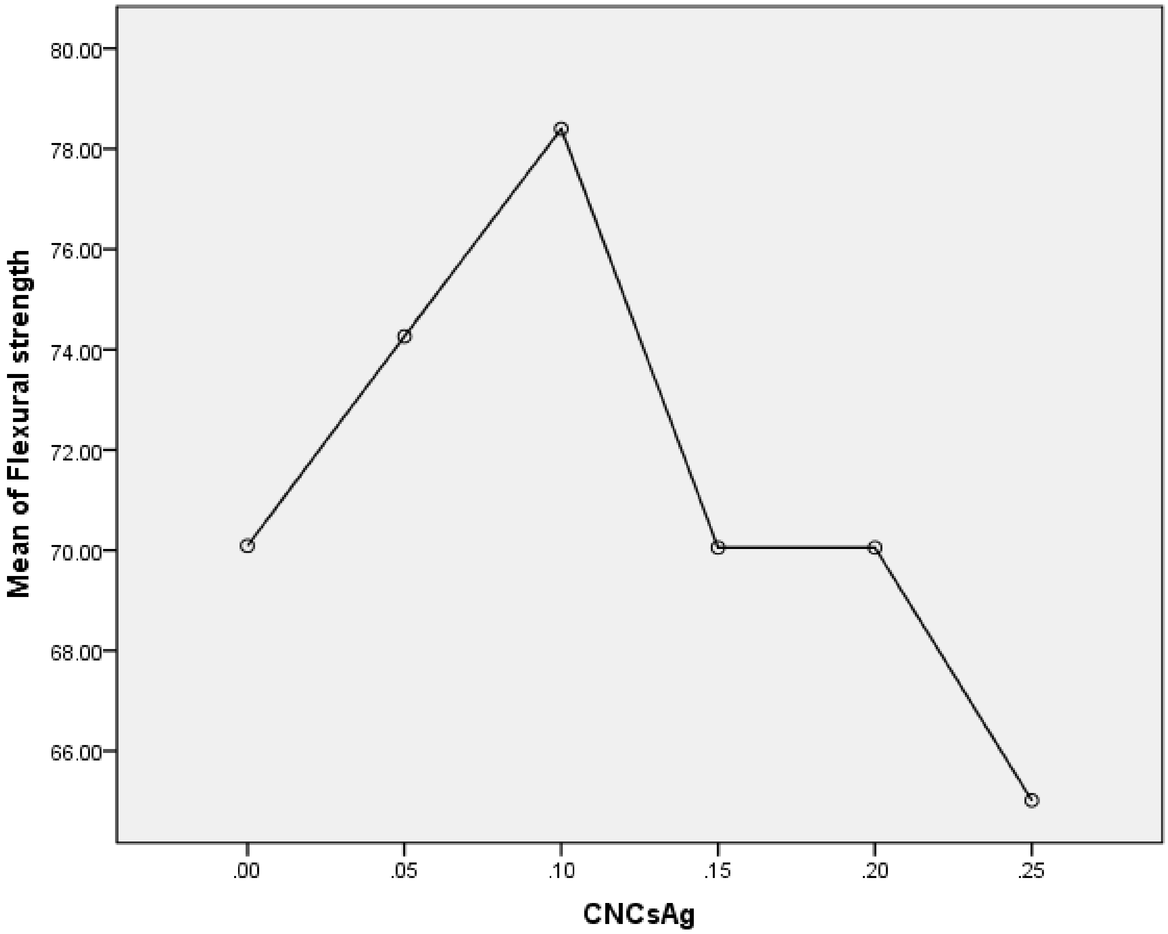

ONEWAY VAR00007 BY VAR00005
/STATISTICS DESCRIPTIVES HOMOGENEITY
/PLOT MEANS
/MISSING ANALYSIS
/POSTHOC=BONFERRONI ALPHA(0.05).

Oneway

Notes

Output Created		22-NOV-2018 11:35:01
Comments		
Input	Data	C:\Users\Beogene\Desktop\2.sav
	Active Dataset	
	Filter	<none>
	Weight	<none>
	Split File	<none>
	N of Rows in Working Data File	36
Missing Value Handling	Definition of Missing	User-defined missing values are treated as missing.
	Cases Used	Statistics for each analysis are based on cases with no missing data for any variable in the analysis.
Syntax		ONEWAY VAR00007 BY VAR00005 /STATISTICS DESCRIPTIVES HOMOGENEITY /PLOT MEANS /MISSING ANALYSIS /POSTHOC=BONFERRONI ALPHA(0.05).
Resources	Processor Time	00:00:00.53
	Elapsed Time	00:00:00.51

Descriptives

Flexural modulus

	N	Mean	Std. Deviation	Std. Error	95% Confidence Interval for Mean Lower Bound	95% Confidence Interval for Mean Upper Bound
.00	5	2171.8740	234.86084	105.03296	1880.2558	2463.4922
.05	5	2136.1600	88.43433	39.54904	2026.3543	2245.9657
.10	5	2295.0880	196.29850	87.78736	2051.3512	2538.8248
.15	5	2342.5120	55.19920	24.68583	2273.9731	2411.0509
.20	5	2364.0940	86.59940	38.72843	2256.5666	2471.6214
.25	5	2235.6200	102.79863	45.97295	2107.9786	2363.2614
Total	30	2257.5580	155.71744	28.42999	2199.4122	2315.7038

Descriptives

Flexural modulus

	Minimum	Maximum
.00	1959.84	2495.73
.05	2071.93	2290.41
.10	2079.66	2606.21
.15	2282.96	2410.38
.20	2283.65	2499.39
.25	2125.26	2359.20
Total	1959.84	2606.21

Test of Homogeneity of Variances

Flexural modulus

Levene Statistic	df1	df2	Sig.
3.649	5	24	.013

ANOVA

Flexural modulus

	Sum of Squares	df	Mean Square	F	Sig.
Between Groups	212680.502	5	42536.100	2.081	.103
Within Groups	490509.254	24	20437.886		
Total	703189.756	29			

Post Hoc Tests

Multiple Comparisons

Dependent Variable: Flexural modulus

Bonferroni

(I) CNCsAg	(J) CNCsAg	Mean Difference (I-J)	Std. Error	Sig.	95% Confidence Interval	
					Lower Bound	Upper Bound
.00	.05	35.71400	90.41656	1.000	-258.8977	330.3257

	.10	-123.21400	90.41656	1.000	-417.8257	171.3977
	.15	-170.63800	90.41656	1.000	-465.2497	123.9737
	.20	-192.22000	90.41656	.660	-486.8317	102.3917
	.25	-63.74600	90.41656	1.000	-358.3577	230.8657
.05	.00	-35.71400	90.41656	1.000	-330.3257	258.8977
	.10	-158.92800	90.41656	1.000	-453.5397	135.6837
	.15	-206.35200	90.41656	.474	-500.9637	88.2597
	.20	-227.93400	90.41656	.281	-522.5457	66.6777
	.25	-99.46000	90.41656	1.000	-394.0717	195.1517
.10	.00	123.21400	90.41656	1.000	-171.3977	417.8257
	.05	158.92800	90.41656	1.000	-135.6837	453.5397
	.15	-47.42400	90.41656	1.000	-342.0357	247.1877
	.20	-69.00600	90.41656	1.000	-363.6177	225.6057
	.25	59.46800	90.41656	1.000	-235.1437	354.0797
.15	.00	170.63800	90.41656	1.000	-123.9737	465.2497
	.05	206.35200	90.41656	.474	-88.2597	500.9637
	.10	47.42400	90.41656	1.000	-247.1877	342.0357
	.20	-21.58200	90.41656	1.000	-316.1937	273.0297
	.25	106.89200	90.41656	1.000	-187.7197	401.5037
.20	.00	192.22000	90.41656	.660	-102.3917	486.8317
	.05	227.93400	90.41656	.281	-66.6777	522.5457
	.10	69.00600	90.41656	1.000	-225.6057	363.6177
	.15	21.58200	90.41656	1.000	-273.0297	316.1937
	.25	128.47400	90.41656	1.000	-166.1377	423.0857
.25	.00	63.74600	90.41656	1.000	-230.8657	358.3577
	.05	99.46000	90.41656	1.000	-195.1517	394.0717
	.10	-59.46800	90.41656	1.000	-354.0797	235.1437
	.15	-106.89200	90.41656	1.000	-401.5037	187.7197
	.20	-128.47400	90.41656	1.000	-423.0857	166.1377

Means Plots

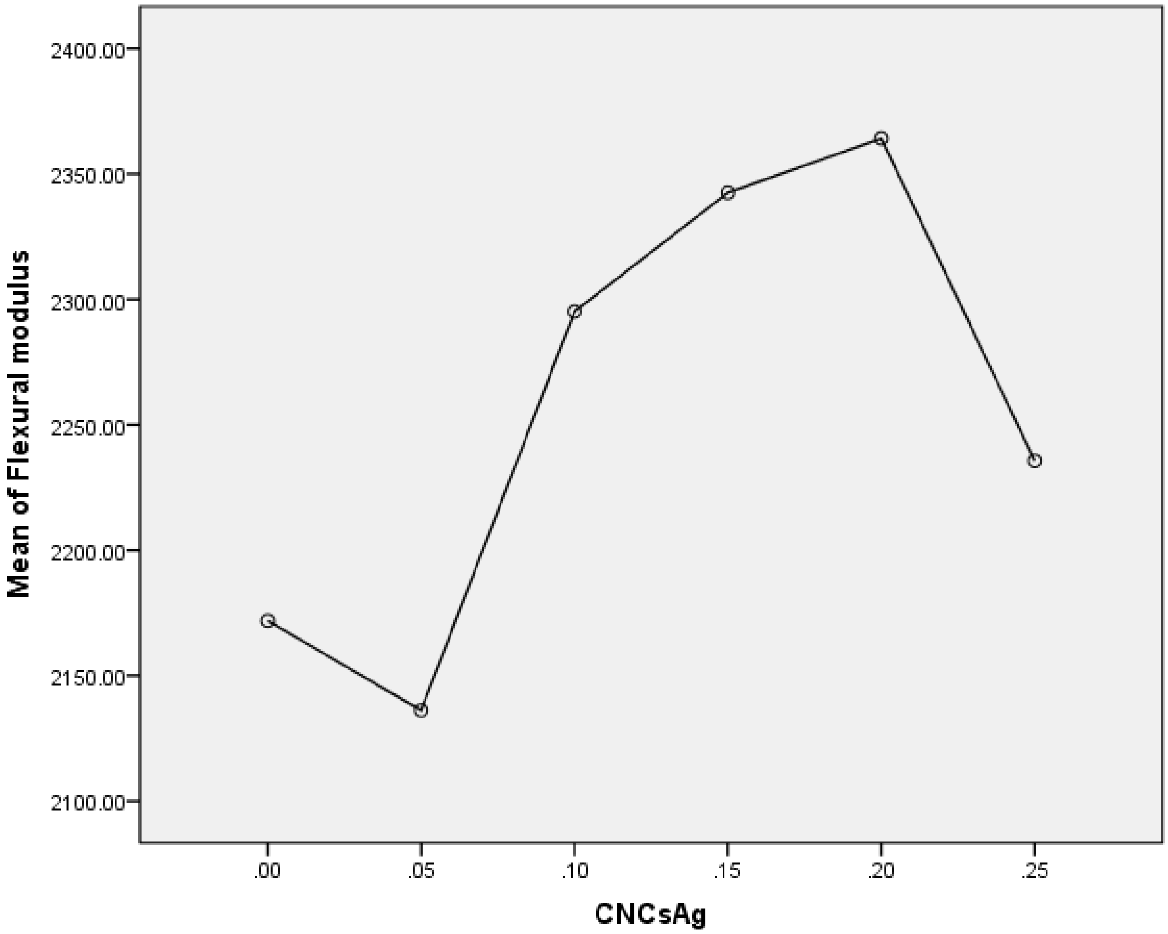

2400.00
2350.00
2300.00
2250.00
2200.00
2150.00
2100.00
Mean of Flexural modulus
.00
.05
.10
.15
.20
.25
CNCsAg
