# Supplementary material for: A Study of 3D-Printable Reinforced Composite Resin: PMMA Modified with Silver Nanoparticles Loaded Cellulose Nanocrystal
Source: Materials (Basel). 2018 Dec 3;11(12):2444. doi: 10.3390/ma11122444 (PMC6317163; doi:10.3390/ma11122444)

```
ONEWAY survivalrateD1 BY CNCsAg
  /STATISTICS DESCRIPTIVES HOMOGENEITY
  /PLOT MEANS
  /MISSING ANALYSIS
  /POSTHOC=BONFERRONI ALPHA(0.05).
```

Oneway

Descriptives

survivalrateD1

	N	Mean	Std. Deviation	Std. Error	95% Confidence Interval for Mean	
					Lower Bound	Upper Bound
.00	3	.9984	.06370	.03678	.8401	1.1566
.02	3	1.0413	.04777	.02758	.9227	1.1600
.05	3	1.0003	.11638	.06719	.7112	1.2894
.10	3	1.0587	.07180	.04145	.8803	1.2371
.15	3	1.0404	.06606	.03814	.8763	1.2045
.25	3	1.0262	.03980	.02298	.9273	1.1251
Total	18	1.0276	.06450	.01520	.9955	1.0596

Descriptives

survivalrateD1

	Minimum	Maximum
.00	.95	1.07
.02	.99	1.09
.05	.92	1.13
.10	.99	1.14
.15	.97	1.11
.25	.98	1.06
Total	.92	1.14

Test of Homogeneity of Variances

survivalrateD1

Levene Statistic	df1	df2	Sig.
1.393	5	12	.294

ANOVA

survivalrateD1

	Sum of Squares	df	Mean Square	F	Sig.
Between Groups	.009	5	.002	.339	.879
Within Groups	.062	12	.005		
Total	.071	17			

Post Hoc Tests

Multiple Comparisons

Dependent Variable: survivalrateD1

Bonferroni

(I) CNCsAg	(J) CNCsAg	Mean Difference (I-J)	Std. Error	Sig.	95% Confidence Interval Lower Bound	Upper Bound
.00	.02	-.04297	.05868	1.000	-.2571	.1711
	.05	-.00196	.05868	1.000	-.2161	.2121
	.10	-.06033	.05868	1.000	-.2744	.1538
	.15	-.04207	.05868	1.000	-.2562	.1720
	.25	-.02783	.05868	1.000	-.2419	.1863
.02	.00	.04297	.05868	1.000	-.1711	.2571
	.05	.04101	.05868	1.000	-.1731	.2551
	.10	-.01736	.05868	1.000	-.2315	.1967
	.15	.00090	.05868	1.000	-.2132	.2150
	.25	.01513	.05868	1.000	-.1990	.2292
.05	.00	.00196	.05868	1.000	-.2121	.2161
	.02	-.04101	.05868	1.000	-.2551	.1731
	.10	-.05837	.05868	1.000	-.2725	.1557
	.15	-.04011	.05868	1.000	-.2542	.1740
	.25	-.02588	.05868	1.000	-.2400	.1882
.10	.00	.06033	.05868	1.000	-.1538	.2744
	.02	.01736	.05868	1.000	-.1967	.2315

	.05	.05837	.05868	1.000	-.1557	.2725
	.15	.01826	.05868	1.000	-.1958	.2324
	.25	.03249	.05868	1.000	-.1816	.2466
.15	.00	.04207	.05868	1.000	-.1720	.2562
	.02	-.00090	.05868	1.000	-.2150	.2132
	.05	.04011	.05868	1.000	-.1740	.2542
	.10	-.01826	.05868	1.000	-.2324	.1958
	.25	.01424	.05868	1.000	-.1999	.2283
.25	.00	.02783	.05868	1.000	-.1863	.2419
	.02	-.01513	.05868	1.000	-.2292	.1990
	.05	.02588	.05868	1.000	-.1882	.2400
	.10	-.03249	.05868	1.000	-.2466	.1816
	.15	-.01424	.05868	1.000	-.2283	.1999

Means Plots

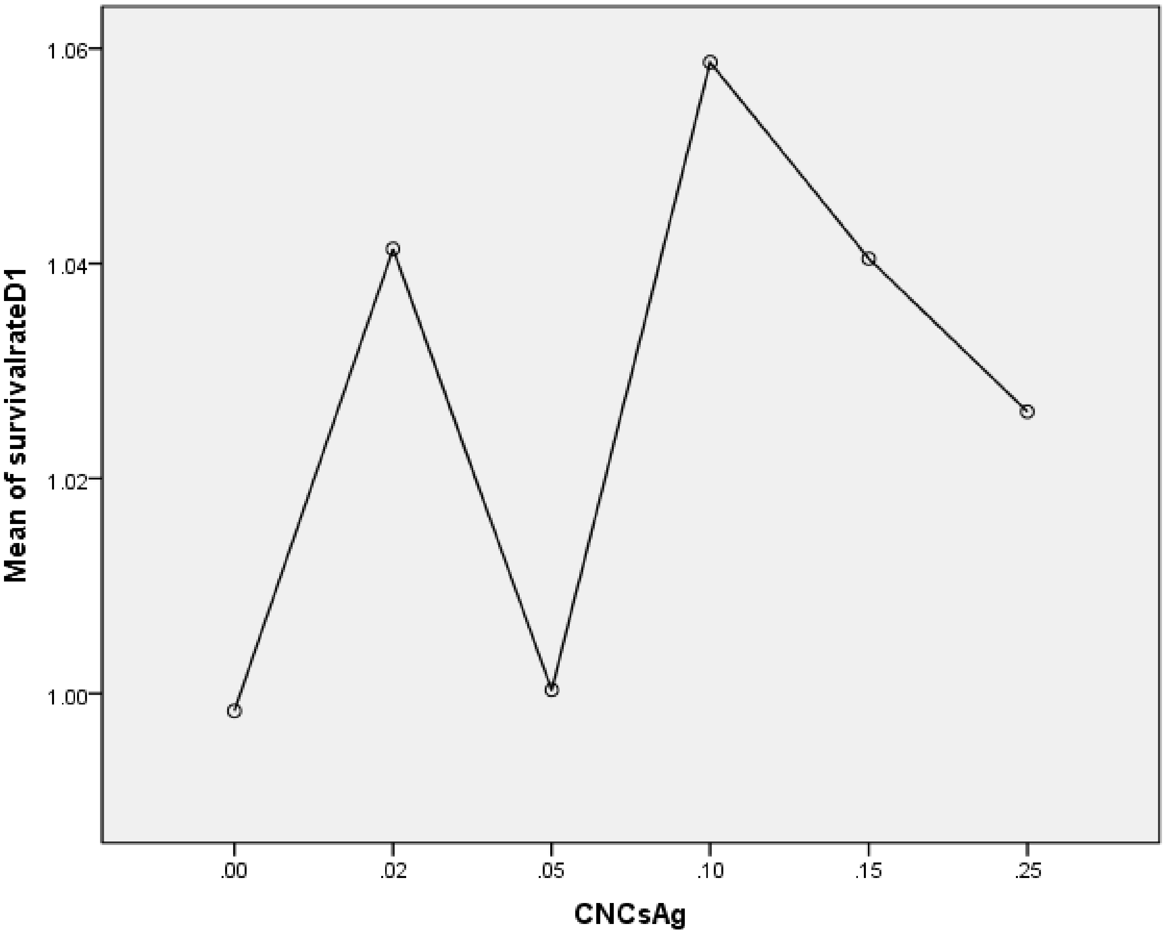
1.06
1.04
1.02
1.00
Mean of survivalrateD1
.00
.02
.05
.10
.15
.25
CNCsAg

```
ONEWAY survivalrateD3 BY CNCsAg
  /STATISTICS DESCRIPTIVES HOMOGENEITY
  /PLOT MEANS
  /MISSING ANALYSIS
  /POSTHOC=BONFERRONI ALPHA(0.05).
```

Oneway

Descriptives

survivalrateD3

	N	Mean	Std. Deviation	Std. Error	95% Confidence Interval for Mean	
					Lower Bound	Upper Bound
.00	3	.9936	.07600	.04388	.8048	1.1824
.02	3	.9398	.07557	.04363	.7520	1.1275
.05	3	.9433	.04074	.02352	.8421	1.0446
.10	3	.9843	.01872	.01081	.9378	1.0308
.15	3	.9753	.08736	.05044	.7583	1.1923
.25	3	.8985	.01445	.00834	.8626	.9344
Total	18	.9558	.06021	.01419	.9259	.9857

Descriptives

survivalrateD3

	Minimum	Maximum
.00	.92	1.07
.02	.89	1.03
.05	.90	.98
.10	.96	1.00
.15	.90	1.07
.25	.88	.91
Total	.88	1.07

Test of Homogeneity of Variances

survivalrateD3

Levene Statistic	df1	df2	Sig.
1.945	5	12	.160

ANOVA

survivalrateD3

	Sum of Squares	df	Mean Square	F	Sig.
Between Groups	.019	5	.004	1.066	.426
Within Groups	.043	12	.004		
Total	.062	17			

Post Hoc Tests

Multiple Comparisons

Dependent Variable: survivalrateD3

Bonferroni

(I) CNCsAg	(J) CNCsAg	Mean Difference (I-J)	Std. Error	Sig.	95% Confidence Interval Lower Bound	Upper Bound
.00	.02	.05380	.04869	1.000	-.1239	.2315
	.05	.05022	.04869	1.000	-.1274	.2279
	.10	.00921	.04869	1.000	-.1685	.1869
	.15	.01824	.04869	1.000	-.1594	.1959
	.25	.09507	.04869	1.000	-.0826	.2727
.02	.00	-.05380	.04869	1.000	-.2315	.1239
	.05	-.00358	.04869	1.000	-.1812	.1741
	.10	-.04459	.04869	1.000	-.2223	.1331
	.15	-.03555	.04869	1.000	-.2132	.1421
	.25	.04127	.04869	1.000	-.1364	.2189
.05	.00	-.05022	.04869	1.000	-.2279	.1274
	.02	.00358	.04869	1.000	-.1741	.1812
	.10	-.04101	.04869	1.000	-.2187	.1367
	.15	-.03197	.04869	1.000	-.2096	.1457
	.25	.04485	.04869	1.000	-.1328	.2225
.10	.00	-.00921	.04869	1.000	-.1869	.1685
	.02	.04459	.04869	1.000	-.1331	.2223
	.05	.04101	.04869	1.000	-.1367	.2187
	.15	.00904	.04869	1.000	-.1686	.1867
	.25	.08586	.04869	1.000	-.0918	.2635
.15	.00	-.01824	.04869	1.000	-.1959	.1594
	.02	.03555	.04869	1.000	-.1421	.2132

	.05	.03197	.04869	1.000	-.1457	.2096
	.10	-.00904	.04869	1.000	-.1867	.1686
	.25	.07682	.04869	1.000	-.1008	.2545
.25	.00	-.09507	.04869	1.000	-.2727	.0826
	.02	-.04127	.04869	1.000	-.2189	.1364
	.05	-.04485	.04869	1.000	-.2225	.1328
	.10	-.08586	.04869	1.000	-.2635	.0918
	.15	-.07682	.04869	1.000	-.2545	.1008

Means Plots

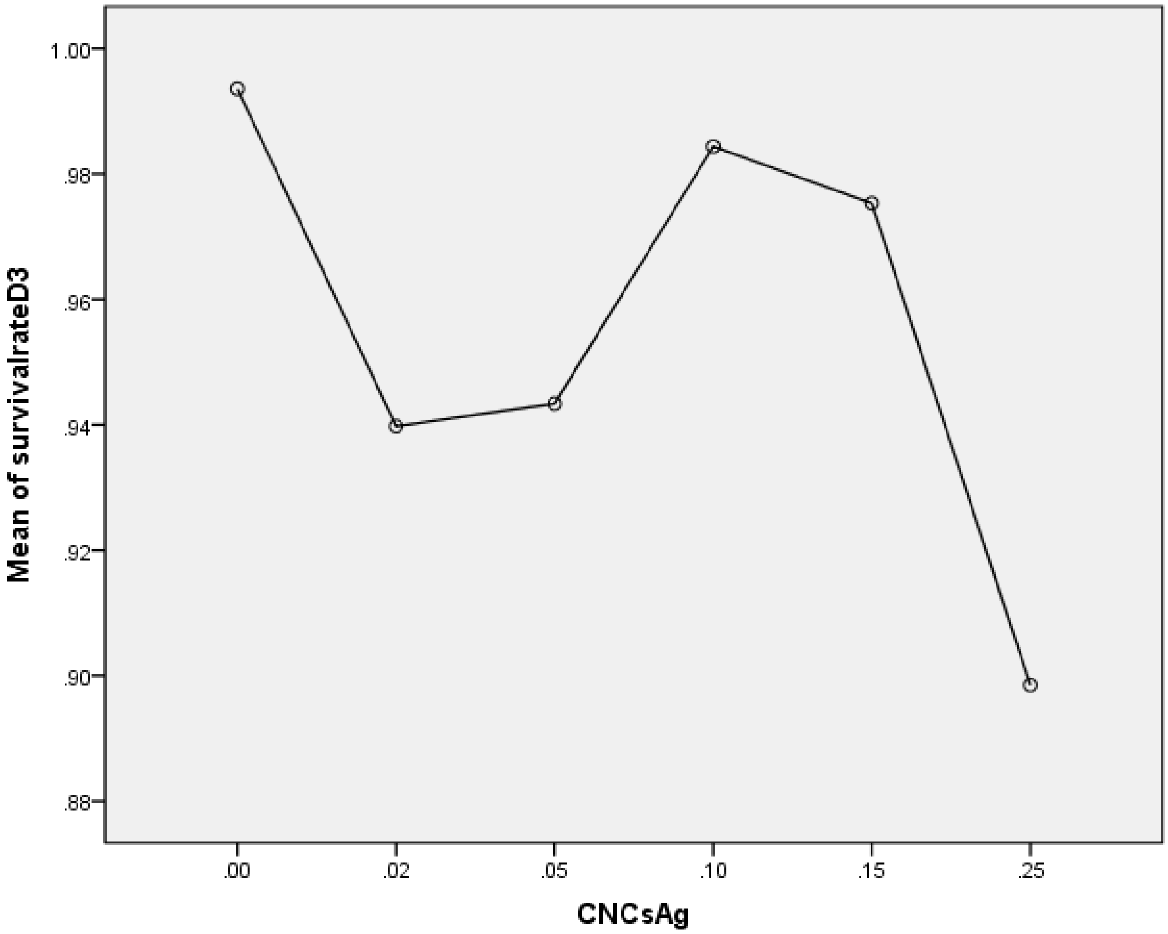

```
ONEWAY survivalrateD5 BY CNCsAg
  /STATISTICS DESCRIPTIVES HOMOGENEITY
  /PLOT MEANS
  /MISSING ANALYSIS
  /POSTHOC=BONFERRONI ALPHA(0.05).
```

Oneway

Descriptives

survivalrateD5

	N	Mean	Std. Deviation	Std. Error	95% Confidence Interval for Mean	
					Lower Bound	Upper Bound
.00	3	.9916	.04304	.02485	.8847	1.0985
.02	3	.9652	.11431	.06600	.6813	1.2492
.05	3	1.0347	.04181	.02414	.9308	1.1386
.10	3	.9204	.04147	.02394	.8174	1.0234
.15	3	.9384	.04376	.02527	.8297	1.0472
.25	3	.9309	.01694	.00978	.8888	.9730
Total	18	.9636	.06390	.01506	.9318	.9953

Descriptives

survivalrateD5

	Minimum	Maximum
.00	.95	1.03

.02	.86	1.09
.05	1.00	1.08
.10	.87	.95
.15	.91	.99
.25	.91	.94
Total	.86	1.09

Test of Homogeneity of Variances

survivalrateD5

Levene Statistic	df1	df2	Sig.
2.056	5	12	.142

ANOVA

survivalrateD5

	Sum of Squares	df	Mean Square	F	Sig.
Between Groups	.028	5	.006	1.645	.222
Within Groups	.041	12	.003		
Total	.069	17			

Post Hoc Tests

Multiple Comparisons

Dependent Variable: survivalrateD5

Bonferroni

(I) CNCsAg	(J) CNCsAg	Mean Difference (I-J)	Std. Error	Sig.	95% Confidence Interval	
					Lower Bound	Upper Bound
.00	.02	.02639	.04783	1.000	-.1481	.2009
	.05	-.04309	.04783	1.000	-.2176	.1314
	.10	.07119	.04783	1.000	-.1033	.2457
	.15	.05318	.04783	1.000	-.1213	.2277
	.25	.06072	.04783	1.000	-.1138	.2352
.02	.00	-.02639	.04783	1.000	-.2009	.1481
	.05	-.06948	.04783	1.000	-.2440	.1050
	.10	.04480	.04783	1.000	-.1297	.2193
	.15	.02678	.04783	1.000	-.1477	.2013
	.25	.03433	.04783	1.000	-.1402	.2089
.05	.00	.04309	.04783	1.000	-.1314	.2176
	.02	.06948	.04783	1.000	-.1050	.2440
	.10	.11428	.04783	.513	-.0602	.2888
	.15	.09626	.04783	1.000	-.0783	.2708

	.25	.10381	.04783	.761	-.0707	.2783
.10	.00	-.07119	.04783	1.000	-.2457	.1033
	.02	-.04480	.04783	1.000	-.2193	.1297
	.05	-.11428	.04783	.513	-.2888	.0602
	.15	-.01802	.04783	1.000	-.1925	.1565
	.25	-.01047	.04783	1.000	-.1850	.1641
.15	.00	-.05318	.04783	1.000	-.2277	.1213
	.02	-.02678	.04783	1.000	-.2013	.1477
	.05	-.09626	.04783	1.000	-.2708	.0783
	.10	.01802	.04783	1.000	-.1565	.1925
	.25	.00755	.04783	1.000	-.1670	.1821
.25	.00	-.06072	.04783	1.000	-.2352	.1138
	.02	-.03433	.04783	1.000	-.2089	.1402
	.05	-.10381	.04783	.761	-.2783	.0707
	.10	.01047	.04783	1.000	-.1641	.1850
	.15	-.00755	.04783	1.000	-.1821	.1670

Means Plots

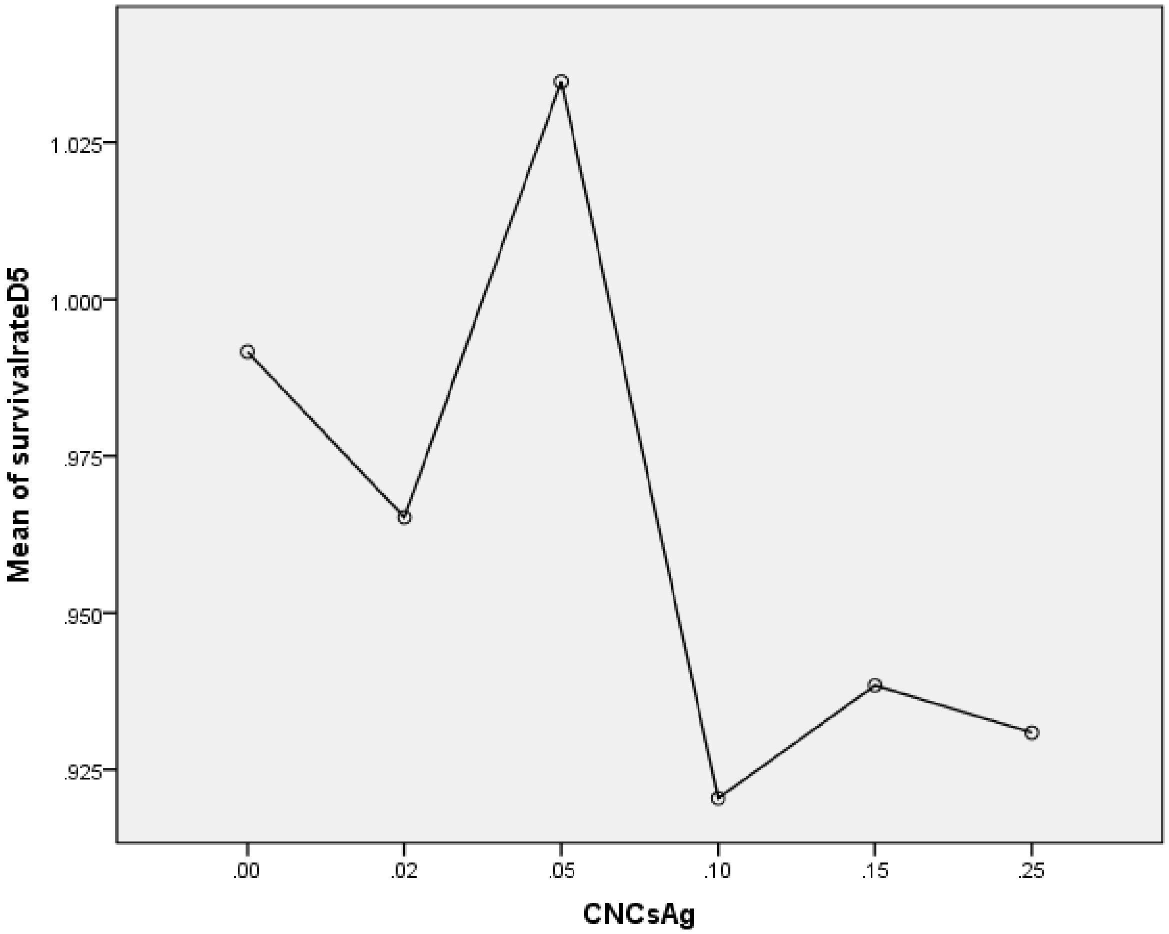

Mean of survivalrateD5
1.025
1.000
.975
.950
.925
.00
.02
.05
.10
.15
.25
CNCsAg

```
ONEWAY survivalrateD7 BY CNCsAg
  /STATISTICS DESCRIPTIVES HOMOGENEITY
  /PLOT MEANS
  /MISSING ANALYSIS
  /POSTHOC=BONFERRONI ALPHA(0.05).
```

Oneway

Descriptives

survivalrateD7

	N	Mean	Std. Deviation	Std. Error	95% Confidence Interval for Mean	
					Lower Bound	Upper Bound
.00	3	.9498	.05066	.02925	.8240	1.0756
.02	3	.9897	.04407	.02544	.8802	1.0992
.05	3	.9617	.02038	.01177	.9111	1.0123
.10	3	.9383	.09723	.05614	.6967	1.1798
.15	3	.9204	.02689	.01552	.8536	.9872
.25	3	.9383	.04565	.02636	.8249	1.0517
Total	18	.9497	.05027	.01185	.9247	.9747

Descriptives

survivalrateD7

	Minimum	Maximum
.00	.90	1.00
.02	.96	1.04
.05	.94	.97
.10	.88	1.05
.15	.89	.94
.25	.89	.97
Total	.88	1.05

Test of Homogeneity of Variances

survivalrateD7

Levene Statistic	df1	df2	Sig.
3.199	5	12	.046

ANOVA

survivalrateD7

	Sum of Squares	df	Mean Square	F	Sig.
Between Groups	.009	5	.002	.599	.702
Within Groups	.034	12	.003		
Total	.043	17			

Post Hoc Tests

Multiple Comparisons

Dependent Variable: survivalrateD7

Bonferroni

(I) CNCsAg	(J) CNCsAg	Mean Difference (I-J)	Std. Error	Sig.	95% Confidence Interval	
					Lower Bound	Upper Bound
.00	.02	-.03991	.04370	1.000	-.1994	.1195
	.05	-.01189	.04370	1.000	-.1713	.1476
	.10	.01152	.04370	1.000	-.1479	.1710
	.15	.02936	.04370	1.000	-.1301	.1888
	.25	.01151	.04370	1.000	-.1479	.1710
.02	.00	.03991	.04370	1.000	-.1195	.1994
	.05	.02802	.04370	1.000	-.1314	.1875
	.10	.05143	.04370	1.000	-.1080	.2109
	.15	.06926	.04370	1.000	-.0902	.2287
	.25	.05142	.04370	1.000	-.1080	.2109
.05	.00	.01189	.04370	1.000	-.1476	.1713
	.02	-.02802	.04370	1.000	-.1875	.1314
	.10	.02341	.04370	1.000	-.1360	.1829
	.15	.04124	.04370	1.000	-.1182	.2007
	.25	.02340	.04370	1.000	-.1360	.1828
.10	.00	-.01152	.04370	1.000	-.1710	.1479
	.02	-.05143	.04370	1.000	-.2109	.1080
	.05	-.02341	.04370	1.000	-.1829	.1360
	.15	.01784	.04370	1.000	-.1416	.1773
	.25	-.00001	.04370	1.000	-.1595	.1594
.15	.00	-.02936	.04370	1.000	-.1888	.1301
	.02	-.06926	.04370	1.000	-.2287	.0902

	.05	-.04124	.04370	1.000	-.2007	.1182
	.10	-.01784	.04370	1.000	-.1773	.1416
	.25	-.01784	.04370	1.000	-.1773	.1416
.25	.00	-.01151	.04370	1.000	-.1710	.1479
	.02	-.05142	.04370	1.000	-.2109	.1080
	.05	-.02340	.04370	1.000	-.1828	.1360
	.10	.00001	.04370	1.000	-.1594	.1595
	.15	.01784	.04370	1.000	-.1416	.1773

Means Plots

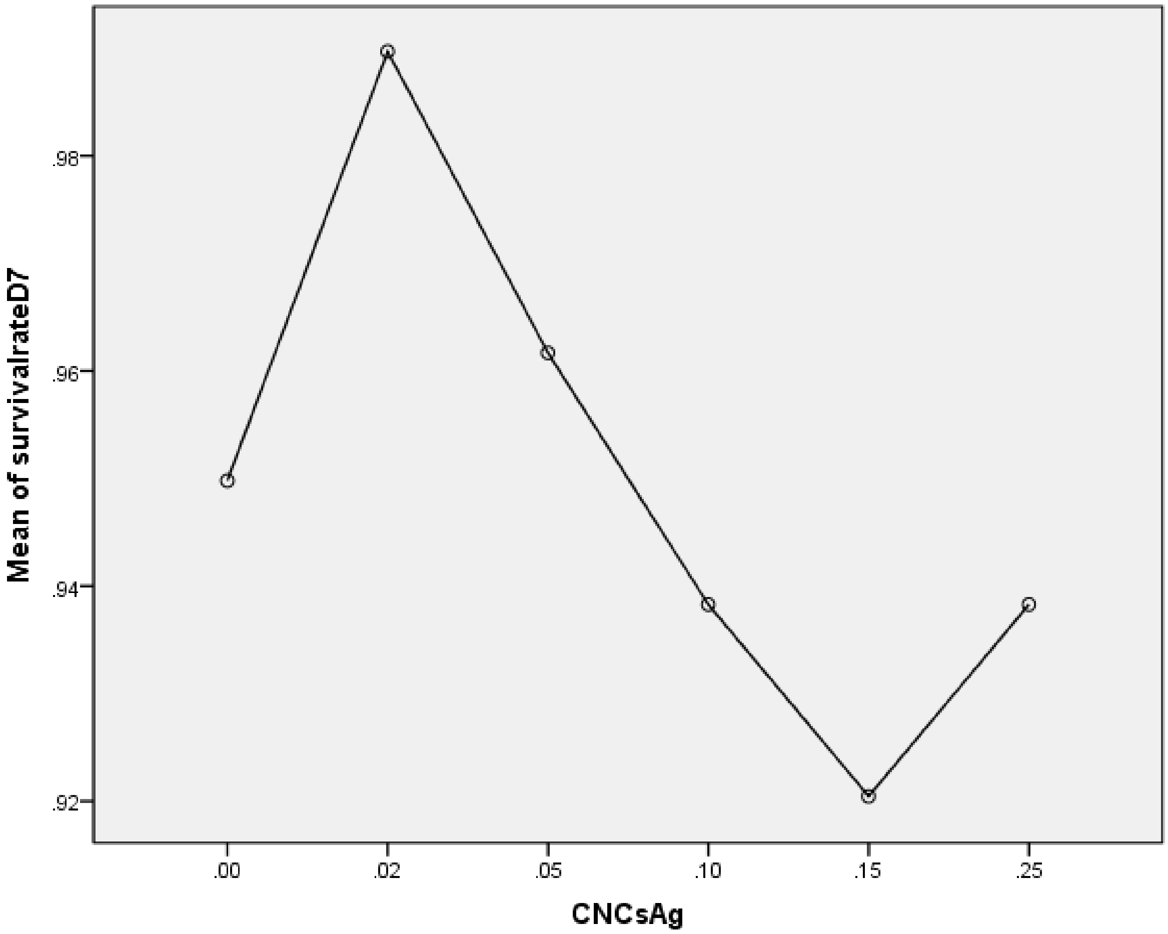

Supplement: Supplementary file 1 [file materials-11-02444-s001.zip › anova fig6.pdf]
